# Functional characterization of VirB/VirD4 and Icm/Dot type IV secretion systems from the plant-pathogenic bacterium *Xanthomonas euvesicatoria*

**DOI:** 10.3389/fcimb.2023.1203159

**Published:** 2023-08-01

**Authors:** Sabine Drehkopf, Felix Scheibner, Daniela Büttner

**Affiliations:** Institute for Biology, Department of Genetics, Martin-Luther University Halle-Wittenberg, Halle (Saale), Germany

**Keywords:** Type IV secretion, conjugation, Icm/Dot, VirD4, plant pathogen, effector, relaxase

## Abstract

**Introduction:**

Many Gram-negative plant- and animal-pathogenic bacteria employ type IV secretion (T4S) systems to transport proteins or DNA/protein complexes into eukaryotic or bacterial target cells. T4S systems have been divided into minimized and expanded T4S systems and resemble the VirB/VirD4 T4S system from the plant pathogen *Agrobacterium tumefaciens* and the Icm/Dot T4S system from the human pathogen *Legionella pneumophila*, respectively. The only known plant pathogen with both types of T4S systems is *Xanthomonas euvesicatoria* which is the causal agent of bacterial spot disease on pepper and tomato plants.

**Results and discussion:**

In the present study, we show that *virB/virD4* and *icm/dot* T4S genes are expressed and encode components of oligomeric complexes corresponding to known assemblies of VirB/VirD4 and Icm/Dot proteins. Both T4S systems are dispensable for the interaction of *X. euvesicatoria* with its host plants and do not seem to confer contact-dependent lysis of other bacteria, which was previously shown for the chromosomally encoded VirB/VirD4 T4S system from *Xanthomonas axonopodis* pv. *citri.* The corresponding chromosomal T4S gene cluster from *X. euvesicatoria* is incomplete, however, the second plasmid-localized *vir* gene cluster encodes a functional VirB/VirD4 T4S system which contributes to plasmid transfer. In agreement with this finding, we identified the predicted relaxase TraI as substrate of the T4S systems from *X. euvesicatoria*. TraI and additional candidate T4S substrates with homology to T4S effectors from *X. axonopodis* pv. *citri* interact with the T4S coupling protein VirD4. Interestingly, however, the predicted C-terminal VirD4-binding sites are not sufficient for T4S, suggesting the contribution of additional yet unknown mechanisms to the targeting of T4S substrates from *X. euvesicatoria* to both VirB/VirD4 and Icm/Dot T4S systems.

## Introduction

Gram-negative plant- and animal-pathogenic bacteria employ at least six types of protein secretion systems for the delivery of proteins or protein/DNA complexes into the extracellular milieu or into bacterial or eukaryotic target cells (Costa et al., 2015; Galan and Waksman, 2018). Essential for pathogenicity is often the type III secretion (T3S) system, which translocates type III effector (T3E) proteins into eukaryotic cells to manipulate metabolic pathways to the bacterial benefit (Büttner, 2012; Wagner et al., 2018; Bastedo et al., 2020; Ceulemans et al., 2021). Alternatively, some pathogens employ type IV secretion (T4S) or type VI secretion systems to translocate DNA/protein complexes or effector proteins into eukaryotic cells (Galan and Waksman, 2018). T4S systems are highly versatile nanomachines which are grouped into conjugation systems, effector translocators and contact-independent uptake or release systems (Christie, 2016; Li et al., 2019; Waksman, 2019). Conjugation systems are the largest group of T4S systems and one of the major drivers of interbacterial exchange of mobile genetic elements including antibiotic resistance and virulence genes (Christie et al., 2017; Li et al., 2019). T4S system-dependent genetic exchange allows the bacterial acquisition of novel traits, thus potentially resulting in more virulent and antibiotic-resistant strains and posing a major threat on human and animal health (Soucy et al., 2015; Shen et al., 2022).

Bacterial pathogens, which depend on T4S systems for successful infection of their host organisms, include e.g., the human pathogen *Legionella pneumophila* or the plant pathogen *Agrobacterium tumefaciens* (Kubori and Nagai, 2016; Li et al., 2019). The VirB/VirD4 T4S system from *A. tumefaciens* transports T-DNA (transfer DNA) and proteins into plant cells and is similar to bacterial conjugation systems (Christie, 2016; Li and Christie, 2018). VirB/VirD4-like T4S systems consist of 12 subunits including VirB1 – VirB11 and VirD4 and represent the prototype of minimized T4S systems. The designation “minimized T4S system” refers to any bacterial T4S system with a similar number of components than the VirB/VirD4 T4S system. In contrast, more complex T4S systems are grouped as “expanded” T4S systems and include e.g., the Icm/Dot (intracellular multiplication/defect in organelle trafficking) T4S system from *L. pneumophila* (Christie et al., 2017; Costa et al., 2021). The Icm/Dot T4S system is one of the largest known T4S systems and is assembled by more than 25 components including several counterparts of VirB/VirD4 proteins (Kitao et al., 2022; Sheedlo et al., 2022).

Both VirB/VirD4- and Icm/Dot-like T4S systems consist of heterooligomeric protein complexes which span both bacterial membranes and are associated with an extracellular pilus (Li et al., 2019; Sheedlo et al., 2022). Cryo-electron tomography provided structural insights into the architecture of four different T4S systems which were isolated from intact bacteria and include a minimized T4S system from *E. coli* encoded on plasmid pKM101 and expanded T4S systems such as the F plasmid-encoded T4S system, the Cag T4S system from *Helicobacter pylori* and the Icm/Dot T4S system from *L. pneumophila* (Sheedlo et al., 2022). Together with cryo-electron microscopy imaging of isolated T4S system subassemblies, these studies revealed the presence of heterooligomeric protein complexes in the outer membrane (OM) and inner membrane (IM) (Costa et al., 2021; Sheedlo et al., 2022). In VirB/VirD4-like T4S systems, a central core complex in the OM and the periplasm is assembled as two ring-like layers by 14 copies of the C-terminal domain of VirB10 surrounded by VirB7 and VirB9 (Costa et al., 2021; Sheedlo et al., 2022). This OM complex is connected *via* a stalk structure to the IM complex which consists of 12 copies of VirB3, VirB6 and VirB8 associated with 14 copies of the N-terminal region of VirB10 (Costa et al., 2021; Sheedlo et al., 2022). Additional components of the IM complex are the three cytoplasmic ATPases VirB4, VirB11 and VirD4, which are involved in substrate recognition and transport (Costa et al., 2021; Sheedlo et al., 2022).

In expanded T4S systems such as the Icm/Dot T4S system from *L. pneumophila*, the central core complex is assembled by DotD, DotH, and DotG which are functional counterparts of VirB7, VirB9, and VirB10, respectively (Kitao et al., 2022; Sheedlo et al., 2022). Additional proteins specific for Icm/Dot T4S systems are involved in the formation of the membrane-spanning OM and IM complexes including the core complex components DotC and DotF as well as two cytoplasmic ATPases, DotO and DotB, which correspond to VirB4 and VirB11 (Kitao et al., 2022; Sheedlo et al., 2022). Substrate recognition by VirB/VirD4 and Icm/Dot T4S systems depends on the ATPases VirD4 and DotL, respectively, which are referred to as type IV coupling proteins (T4CPs) (Christie et al., 2017; Li et al., 2019; Costa et al., 2021; Meir et al., 2023). The cytoplasmic region of VirD4 contains an alpha-helical bundle, termed all-alpha-domain (AAD), which likely provides a binding site for T4S substrates (Christie et al., 2017; Li et al., 2019; Oka et al., 2022). In Icm/Dot T4S systems, additional adaptor proteins such as IcmS and IcmW are part of a heterooligomeric coupling complex and promote substrate recruitment by DotL (Li et al., 2019; Kitao et al., 2022; Sheedlo et al., 2022). Recognition of cargo proteins depends on different signals including a C-terminal region in T4S substrates from *Xanthomonas axonopodis* pv. *citri*, termed XVIPCD (*Xanthomonas* VirD4-interacting protein conserved domain), which is sequence variable but shares common features such as certain amino acid compositions or structural motifs (Li et al., 2019; Sgro et al., 2019; Costa et al., 2021).

The analysis of T4S systems in Gram-negative bacteria suggests that Icm/Dot T4S systems are predominantly present in human and animal pathogens where they act as important pathogenicity factors in the delivery of effector proteins into eukaryotic host cells (Nagai and Kubori, 2011; Li et al., 2019; Liu and Shin, 2019; Costa et al., 2021). In contrast, plant-pathogenic bacteria such as *A. tumefaciens* depend on VirB/VirD4 T4S systems to interact with their hosts (Li et al., 2019). In *Xanthomonas* spp., VirB/VirD4 T4S systems, also designated Xanthomonadales-like or X-T4S systems, were initially identified as conjugation machines for plasmid transfer (El Yacoubi et al., 2007; Kaur et al., 2019; Sgro et al., 2019). In *X. axonopodis* pv. *citri* and the related human-pathogenic bacterium *Stenotrophomonas maltophilia*, however, VirB/VirD4 T4S systems deliver toxins into bacterial competitor cells (Souza et al., 2015; Bayer-Santos et al., 2019; Sgro et al., 2019). Candidate substrates were identified by yeast two-hybrid analyses as interactors of the T4CP VirD4 which serves as substrate acceptor site (Sgro et al., 2019). The identified *Xanthomonas* VirD4-interacting proteins (XVIPs) include Xanthomonadales-like T4S system effectors (X-Tfes) and share a C-terminal domain of approximately 120 residues with conserved amino acid motifs, designated XVIP conserved domain (XVIPCD) (Alegria et al., 2005; Souza et al., 2015). The XVIPCD presumably provides the binding site for VirD4 and is required for T4S (Souza et al., 2015; Oka et al., 2022).

In the present study, we analyzed the function of T4S systems in *Xanthomonas euvesicatoria*, which is the causal agent of bacterial spot disease in pepper and tomato. *X. euvesciatoria* is the only known plant pathogen with a putative Icm/Dot T4S system and a VirB/VirD4 T4S system. Corresponding genes are located on the chromosome and on plasmids pXCV183 and pXCV38 (Thieme et al., 2005). A possible contribution of T4S systems from *X. euvesicatoria* to the host-pathogen interaction and to conjugation or effector delivery has not yet been investigated. It was previously shown that pathogenicity of *X. euvesicatoria* depends on a T3S system which translocates type III effectors (T3Es) into plant cells and thus allows the manipulation of cellular processes to the advantage of the pathogen (Büttner and Bonas, 2010; Alvarez-Martinez et al., 2021). In addition to T3Es, substrates of the Xps type II secretion (T2S) system contribute to virulence and likely degrade components of the plant cell wall to facilitate the delivery of T3Es (Szczesny et al., 2010; Solé et al., 2015). In the present study, we show that virulence of *X. euvesicatoria* is independent of T4S genes which are expressed *in vitro* and *in planta*. The plasmid-encoded VirB/VirD4 T4S system likely contributes to plasmid transfer between different *X. euvesicatoria* strains, suggesting a role as conjugation machine. Candidate T4S substrates interact with VirD4 and include the putative relaxase TraI and XVIPCD-containing homologs of X-Tfes from *X. axonopodis* pv. *citri*. Interestingly, our data suggests that T4S substrates are targeted to both T4S systems and that the XVIPCD is not sufficient for T4S.

## Materials and methods

### Bacterial strains and growth conditions

Bacterial strains and plasmids used in this study are listed in **Table S1**. *Escherichia coli* strains were grown at 37°C in lysogeny broth (LB) medium, and *X. euvesicatoria* at 30°C in nutrient-yeast extract-glycerol (NYG) medium. Antibiotics were added to the media at the following final concentrations: ampicillin, 100 μg/ml; kanamycin, 25 μg/ml; rifampicin, 100 μg/ml; spectinomycin, 100 μg/ml; streptomycin, 2.5 µg/ml and gentamicin, 15 μg/ml. Plasmids were introduced into *E. coli* by transformation and into *X. euvesicatoria* by electroporation or triparental conjugation, using pRK2013 as helper plasmid.

### Plant materials and plant infections

For infection assays, *X. euvesicatoria* strains were resuspended in 1 mM MgCl_2_ at an optical density (OD_600nm_) of 0.1 which corresponds to 1×10^8^ colony-forming units (CFU) ml^−1^. Bacterial suspensions were infiltrated into leaves of the pepper cultivar Early Cal Wonder (ECW) using a needleless syringe. Infected plants were incubated in growth chambers for 16 hours of light at 28°C and 8 hours of darkness at 22°C. Disease symptoms were photographed seven days post inoculation (dpi). *In planta* growth curves were performed in three ECW plants as described (Bonas et al., 1991). To monitor translocation of dTALE-2, *X. euvesicatoria* strains were resuspended in 1 mM MgCl_2_ at a density of 4×10^8^ CFU ml^−1^ and infiltrated into leaves of *gfp*-transgenic *Nicotiana benthamiana* plants (Werner et al., 2011). Infected *N. benthamiana* plants were incubated for 16 hours of light at 20°C and 8 hours of darkness at 18°C. GFP fluorescence was documented four dpi using a chemiluminescence/fluorescence imager (Vilber Fusion FX Edge). Infection experiments were performed at least three times with different transconjugants; representative results are shown.

### Generation of *X. euvesicatoria* deletion and insertion mutants

To generate *X. euvesicatoria* T4S deletion mutants, DNA fragments flanking individual (*virB4*, *virD4*, *virG*, *virA* and *icmE*) or multiple (*virB11virB1*, *virB6virB8virB9virB10* and *dotCB*) T4S genes were amplified by PCR and cloned into the Golden Gate-compatible suicide vector pOGG2 using the type IIs restriction enzyme *Bsa*I and ligase (Engler et al., 2008). The resulting constructs were transferred into strain 85-10 by triparental conjugation. Transconjugants were selected as described previously and double crossovers resulted in T4S deletion mutant strains (Huguet et al., 1998). Multiple deletions of T4S genes or gene regions resulted from the successive introduction of corresponding deletion constructs into *X. euvesicatoria* strains.

To insert the gentamicin resistance gene into the genome of *X. euvesicatoria* strain 85-10, the gene was amplified by PCR from plasmid pBRM and cloned into the Golden Gate-compatible suicide vector pLAND downstream of the *lac* promoter using *Bsa*I and ligase. pLAND allows the insertion of genes into the *hpaFG* region adjacent to the T3S gene cluster (Lorenz et al., 2012). The corresponding construct pLAND-gent^R^ was introduced into strain 85-10 by conjugation and homologous recombination resulted in strain 85-10::*gent^R^* as described previously (Huguet et al., 1998; Lorenz et al., 2012).

To insert a kanamycin resistance gene and *gfp* (green fluorescent protein) into *X. euvesicatoria*, DNA fragments flanking a non-coding region at nucleotide position 2831 on plasmid pXCV183 were amplified by PCR from strain 85-10. Similarly, the promoter of the gentamicin resistance gene on plasmid pBRM, the kanamycin resistance gene from plasmid pKT25 and the *gfp* gene including the *lac* promoter were amplified by PCR using plasmid pBRM-sfgfp as template. Flanking *Bsa*I sites with matching overhangs were introduced by the primer sequences. The corresponding amplicons were first subcloned in vector pICH41021 using the blunt-end cutter *Sma*I and ligase in a cut-ligation. All five amplicons were subsequently assembled in the Golden Gate-compatible suicide vector pOGG2 using *Bsa*I and ligase. The resulting construct pOGG2-kan^R^gfp(pXCV183) was transferred into strain 85-10 by triparental conjugation. Double crossovers resulted in *X. euvesicatoria* strain 85-10 containing the kanamycin resistance gene and *gfp* on plasmid pXCV183.

### Generation of expression constructs

For the generation of *XCV0332*, *XCV1120* and *XCV3751* expression constructs, genes were amplified by PCR from *X. euvesicatoria* strain 85-10 and individually cloned into the Golden Gate-compatible expression vector pBRNM using *Bsa*I in a single restriction/ligation reaction. Vector pBRNM allows the expression of genes under control of a *lac* promoter in frame with an N-terminal 3 × c-Myc epitope-encoding sequence. To generate the *traI* expression construct, the promoter of *XCV4361*, the 3 × c-Myc epitope-encoding sequence and *traI* were amplified by PCR. Primer sequences introduced *Bsa*I restriction sites with matching overhangs. When compared with the *lac* promoter, the promoter upstream of *XCV4361* results in lower expression levels of downstream genes as was previously shown for the predicted xylanase gene XCV4360 (Solé et al., 2015). All three DNA fragments were assembled by Golden Gate cloning in vector pBRM-P which contains an integrated stop codon downstream of the insert.

For *in cis* expression of *virB4* in *X. euvesicatoria*, *virB4* was amplified by PCR and cloned into the Golden Gate-compatible suicide vector pLAND downstream of the *lac* promoter and in frame with a C-terminal 3 x c-Myc epitope-encoding sequence using *Bsa*I and ligase. To insert *virB4-c-myc* into the genome of *X. euvesicatoria*, pLAND-virB4 was transferred into strain 85-10Δ*virB4*. Double homologous recombination events led to the insertion of *virB4-c-myc* into the *hpaFG* region. For the *in cis* expression of *icmE, icmE* and the promoter of *XCV0160* were amplified by PCR from strain 85-10 and assembled in the Golden Gate-compatible suicide vector pLAND-P in frame with a C-terminal 3 × c-Myc epitope-encoding sequence using *Bsa*I and ligase. The promoter of *XCV0160* was previously shown to mediate gene expression in *X. euvesicatoria* and presumably results in lower expression levels than the constitutive *lac* promoter (Scheibner and Büttner, unpublished data). The resulting construct pLAND-P-icmE was transferred into strain 85-10Δ*icmE* and double homologous recombination events led to the insertion of *icmE-c-myc* into the *hpaFG* region.

For the generation of promoter-reporter constructs, upstream regions of *virB2*, *virB5*, *virD4*, *virG*, *dotA*, *dotD*, *icmL*, *hrpB1* as well as 129 bp downstream of the annotated start codon of *icmL* were amplified by PCR. Each amplicon was ligated with the *sfgfp* reporter gene in the Golden Gate-compatible vector pBRM-P+T, which contains a terminator upstream of the cloning site. The resulting constructs were transferred into *X. euvesicatoria* strains 85-10 and 85-10*ΔvirG*. *vir* and *icm/dot* promoter regions were additionally cloned upstream of the *dTALE-2* (designer transcription activator-like effector 2) gene in a Golden Gate-compatible expression vector using the modular cloning (MoClo) system (Weber et al., 2011). The MoClo system is based on different cloning vectors (designated level -2, -1, 0, 1 and 2 vectors) which allow the stepwise assembly of gene and promoter modules into multigene constructs by the alternate use of *Bsa*I and *Bpi*I restriction enzymes (Weber et al., 2011). *vir* and *icm/dot* promoter regions were first cloned into vector pAGM9121 using *Bpi*I and ligase, thus resulting in level 0 constructs, and were subsequently transferred to vector pICH47732 using *Bsa*I and ligase to create level 1 promoter constructs. For the assembly of *dTALE-2*, modules encoding the N-terminal region (pICH70781), the central repeats (pICH73079, pICH73081, pICH73093) and the C-terminal region of dTALE-2 (pICH72151) were assembled in the level 0 construct pICH73103. The resulting insert was subsequently cloned into vector pICH50251 using *Bsa*I and ligase to create the level 1 dTALE-2 construct pICH79631. For the generation of dTALE-2 expression constructs containing *vir* or *icm/dot* promoters, modules with promoter fragments, the dTALE-2-encoding sequence (pICH79631), and a transcription terminator (pICH50122) were assembled in the level 2 vector pICH77739 using *Bpi*I and ligase.

For bacterial adenylate cyclase-based two-hybrid (BACTH) assays, T4S genes were cloned into the Golden Gate-compatible vectors pUT18GG, pUT18CGG, pKT25GG and pKNT25GG using *Bsa*I and ligase. Additionally, the putative T4S substrate genes *XCV0332*, *traI*, *XCV3751* and *XCV1120* were amplified by PCR from *X. euvesicatoria* strain 85-10, subcloned into pICH41021 as blunt-end fragments using *Sma*I and ligase, and the resulting inserts were cloned into the *Bsa*I sites of vectors pUT18GG, pUT18CGG, pKT25GG and pKNT25GG by Golden Gate cloning. Primers and plasmids used in this study are listed in **Tables S1, S2**.

### *In vitro* secretion experiments and protein analysis

For *in vitro* T4S assays, *X. euvesicatoria* strains were grown over night in NYG medium with antibiotics, resuspended in fresh NYG medium containing 50 μg ml^−1^ bovine serum albumin (BSA) at a cell density of 2 × 10^8^ CFU/ml, and incubated on a rotary shaker at 30°C for 4 h. Bacterial cells and secreted proteins were separated by filtration as described previously (Rossier et al., 1999). Proteins in 2 ml of the culture supernatants were precipitated with trichloroacetic acid and resuspended in 20 μl of Laemmli buffer. Total cell extracts and culture supernatants were analyzed by SDS-PAGE and immunoblotting, using antibodies directed against the c-Myc epitope (Sigma Aldrich) and the β-subunit of the RNA polymerase (Invitrogen), respectively. Horseradish peroxidase-labelled anti-mouse and anti-rabbit antibodies were used as secondary antibodies. Binding of antibodies was visualized using a chemiluminescence imager (Vilber Fusion FX Edge). Results were reproduced at least two times.

### Protein-protein interaction studies using the BACTH system

BACTH assays were performed using the Euromedex BACTH system kit and Golden Gate-compatible expression vectors (**Table S1**). To monitor protein synthesis, expression constructs encoding T25 and T18 fusion proteins were transformed into JM109 *E. coli* cells, and gene expression was induced with IPTG (isopropyl-*β*-D-thiogalactopyranoside; 2 mM final concentration) at an OD_600_ of 0.6 - 0.8. After induction, cultures were incubated on a rotary shaker for 2 h at 37°C. Bacterial cells were collected by centrifugation, resuspended in Laemmli buffer, and analyzed by immunoblotting, using a FLAG epitope-specific antibody.

Protein-protein interaction studies were performed as described previously (Karimova et al., 2005; Otten and Büttner, 2021). Briefly, expression constructs encoding T18- and T25-fusion proteins were cotransformed into chemically competent BTH101 *E. coli* cells and transformants were plated on LB plates containing kanamycin and gentamicin. At least three colonies per transformant were cultivated over night at 30°C on a rotary shaker in liquid LB medium with appropriate antibiotics. Two microliters of each culture were spotted on LB plates containing kanamycin, gentamicin, X-gal (5-bromo-4-chloro-3-indolyl-β-D-galactopyranosid; 40 μg/ml) and 2 mM IPTG. Colonies were photographed over a period of three to 5 days. Every co-transformation was performed at least three times; representative results are shown.

### Competition assays with *X. euvesicatoria* and *E. coli* strains

Growth of *E. coli* during cocultivation with *X. euvesicatoria* was analyzed as described previously (Bayer-Santos et al., 2019). *X. euvesicatoria* and *E. coli* strains were grown overnight in NYG or LB medium, respectively, with appropriate antibiotics. Cells were harvested by centrifugation, resuspended at OD_600nm_ of 0.3 in fresh medium and grown to exponential phase for 4 h on a rotary shaker. The OD_600nm_ of all cultures was measured and adjusted to 1. Serial dilutions (1:2) of *E. coli* cultures were prepared in 96 well plates and equal volumes of *E. coli* and *X. euvesicatoria* cultures were mixed in each well. Five microliters of every suspension were spotted on LB agar plates containing 100 μM IPTG and 40 μg/mL X-gal. Plates were incubated for 24 h at 30˚C and photographed. The experiment was performed three times with similar results.

For quantitative growth competition assays, *X. euvesicatoria* and *E. coli* were cocultivated according to a previously described protocol (Souza et al., 2015). For this, *X. euvesicatoria* strains were grown over night on NYG agar plates containing rifampicin and *E*. *coli* strain Top 10 was grown over night in 5 ml LB liquid medium in the presence of streptinomycin. Cells were resuspended to an OD_600nm_ of 0.2 in liquid NYG or LB medium and cultures were incubated for five hours on a rotary shaker at 30°C for *X. euvesicatoria* or at 37°C for *E. coli*. After incubation, the OD_600nm_ of all cultures was measured and *X. euvesicatoria* was mixed with *E. coli* in triplicates in a final volume of 1 ml at a ratio of 100: 1 (*X. euvesicatoria*: *E.* coli) corresponding to a final OD_600nm_ for *X. euvesicatoria* of 0.05 and for *E. coli* of 0.0005. To measure bacterial growth at 0 hours, we prepared serial dilutions of 100 µl of the mixtures which were plated on LB plates containing streptinomycin for selection of Top 10 and on NYG plates containing rifampicin for selection of *X. euvesicatoria.* For the analysis of bacterial growth after cocultivation of *X. euvesciatoria* and *E. coli*, 100 µl of the mixtures were spotted on LB agar plates and cultivated at 30°C. After 38 hours, bacteria were resuspended in 1 ml of NYG medium and all mixtures were adjusted to the same OD_600nm_. Serial dilutions were subsequently plated on LB plates containing streptinomycin for selection of Top 10 and on NYG plates containing rifampicin for selection of *X. euvesicatoria* and the final *X. euvesicatoria – E. coli* ratios were calculated after counting the colonies.

### Analysis of plasmid transfer in *X. euvesicatoria* strains

Plasmid transfer between *X. euvesicatoria* strains was analyzed according to a protocol by Stall et al. (1986). For this, selected *X. euvesicatoria* strains were grown over night on NYG agar plates containing appropriate antibiotics. Cells were resuspended at an OD_600nm_ of 0.3 in liquid NYG medium without antibiotics. After six hours of incubation at 30°C on a rotary shaker, donor and recipient strains were mixed in 1 ml of NYG medium at a final OD_600nm_ of 0.4 for each strain. Bacterial suspensions were subsequently dropped on a cellulose nitrate filter (NC20, membrane filters, 0.2 µm, diameter 50 mm, Whatman) which was transferred onto an NYG agar plate containing rifampicin. Bacteria were covered with 1 ml 1% water agar and incubated over night at 30°C. Bacterial conjugation mixtures were then transferred from the cellulose nitrate filters to 1 ml of NYG medium using a spatula and adjusted to an OD_600nm_ of 0.05. Serial dilutions were plated on NYG plates (1.5% agar) with appropriate antibiotics. The plates were incubated for two days at 30°C and the transconjugants were counted. The number of transconjugants obtained with the wild-type strain as donor was set to 100%.

### Isolation of plasmids from *X. euvesicatoria*

For the isolation of native plasmids, *X. euvesicatoria* strains were resuspended at an OD_600nm_ of 0.05 in 50 ml NYG medium containing rifampicin and grown over night at 30°C. Plasmids were isolated using the GeneJET plasmid midiprep kit (Thermo Scientific) according to the manufacturer’s instructions. Twenty microliters of each plasmid preparation were loaded on an 0.7% agarose gel containing 5% ethidium bromide. The electrophoresis was performed in 1 x TBE (Tris-borate-EDTA [ethylene diamine tetraacetic acid]) buffer at 120 mA for 25 min and DNA was visualized using an UV imager. The agarose gel electrophoresis was performed three times with similar results.

## Results

### Analysis of *virB/virD4* and *icm/dot* T4S gene clusters in *X. euvesicatoria* strain 85-10

**Figure 1:**
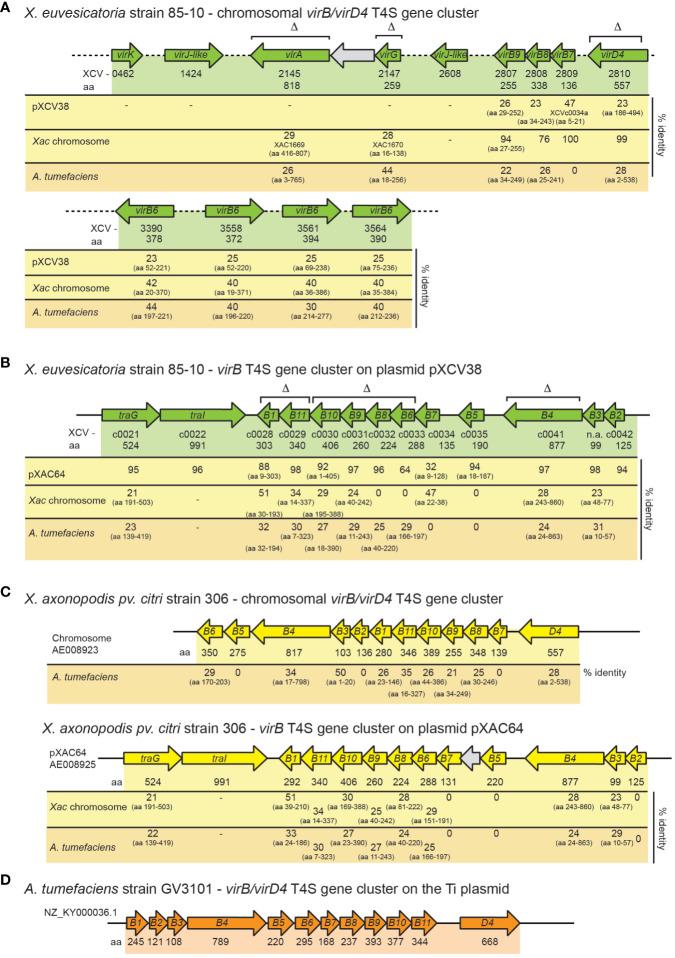
Genetic organization of *vir* gene clusters in *X. euvesicatoria, X. axonopodis* pv. *citri* and *A. tumefaciens.* **(A)** Organization of chromosomal *vir* genes from *X. euvesicatoria* strain 85-10. Genes are represented by arrows. Gene numbers, the length of the corresponding gene products in amino acids (aa) and the percentage of amino acid identity to corresponding proteins encoded on plasmid pXCV38 from *X. euvesicatoria* strain 85-10, on the chromosome of *X. axonopodis* pv. *citri* (*Xac*) strain 306 and of *A. tumefaciens* is indicated. The symbol Δ refers to genes which were deleted in the present study. Accession numbers and predicted functions of all proteins are summarized in **Table S3**. **(B)** Schematic representation of the *vir* gene cluster on plasmid pXCV38 from *X. euvesicatoria* strain 85-10*. virB3* and *virB7* were identified by bioinformatic analyses based on sequence homologies of the corresponding gene products with known VirB3 and VirB7 proteins. Gene numbers, the size of corresponding gene products and the amino acid identity to homologous proteins is indicated as described in **(A)**. **(C)** Organization of the chromosomal and plasmid-localized *vir* gene clusters from *X. axonopodis* pv. *citri* strain 306. Gene numbers, the size of corresponding gene products and the amino acid identity to homologous proteins is indicated as described in **(A)**. **(D)** Schematic representation of the *vir* gene cluster from *A. tumefaciens.* Numbers refer to the size of the corresponding gene products in amino acids.

Previous genome sequence analyses identified predicted *virB/virD4* and *icm/dot* T4S genes on the chromosome and on plasmids pXCV38 and pXCV183 of *X. euvesicatoria* strain 85-10 (Thieme et al., 2005). The chromosomal T4S gene cluster contains *virB7, virB8*, *virB9*, *virD4* and four copies of *virB6* (**Figure 1A**). *virB7, virB8, virB9* and *virD4* genes encode predicted core components of the T4S system which are homologous (76 – 100% amino acid identity) to corresponding proteins from *X. axonopodis* pv. *citri* (**Figure 1A**; **Table S3**). VirB6 proteins are less conserved (40 – 42% amino acid identity) (**Figure 1A**). In contrast to the chromosomally encoded VirB/VirD4 T4S system from *X. axonopodis* pv. *citri*, which was previously shown to deliver toxins into bacterial cells (Souza et al., 2015), the chromosomal *vir* gene cluster from *X. euvesicatoria* is incomplete because it lacks *virB1* to *virB5* as well as *virB10* and *virB11* (**Figure 1A**). Chromosomal *vir* genes of *X. euvesicatoria*, however, include *virA* and *virG* (**Figure 1A**), which encode putative regulators with homology to the corresponding sensor and response regulator of a VirA/VirG two-component system from *A. tumefaciens* (Gelvin, 2003; Wise and Binns, 2015). VirA from *A. tumefaciens* is autophosphorylated and subsequently phosphorylates VirG which binds to *vir* box elements (consensus [5’-TG(A/T)AA(C/T)-3’]) in promoters of target genes (Winans et al., 1987; Jin et al., 1990; Tamamoto et al., 1990; Winans, 1990). Notably, predicted *vir* boxes are located upstream of several *vir* and *icm/dot* genes from *X. euvesicatoria* (**Table 1**).

**Table 1:** Identification of promoters in upstream regions of *vir* and *icm/dot* genes from *X. euvesicatoria* strain 85-10.

Gene	Length of promoter sequence^1^	BROM prediction^2^		*vir* box^3^	GFP synthesis^4^	GFP fluorescence^5^
		-10 region	-35 region			
***virB2***	234	+	+	+	+	+/-
***virB5***	231	+	+	+	+	+
***virD4***	242	–	–	–	+	–
***virG***	301	–	–	–	+	+
***dotA***	240	+	+	+	+	+/-
***dotD***	207	+	+	+	+	+/-
***icmL_229_***	229	–	–	–	–	–
***icmL_129_***	129	+	+	+	+	+

^1^ Putative promoter fragments were cloned upstream of the *sfgfp* gene for expression studies in *X. euvesicatoria* strain 85-10 or upstream of *dTALE*-2 in strain 85-10Δ*xopQ* for the analysis of *in planta* translocation of dTALE-2 into *gfp*-transgenic *N. benthamiana* plants.

^2^ The prediction program BROM (Softberry) http://www.softberry.com/berry.phtml?topic=bprom&group=programs&subgroup=gfindb ) predicts bacterial sigma70 promoter regions.

^3^ *vir* box elements (5’-TG(A/T)AA(C/T)-3’) are present in putative promoter fragments of *vir* and *icm/dot* genes from *X. euvesicatoria*. +, presence of putative *vir* box; -, absence of putative *vir* box.

^4^ GFP synthesis in *X. euvesicatoria* was detected by immunoblot analysis of total cell extracts from bacteria grown in liquid NYG cultures. +, detection of GFP by immunoblotting; -, no GFP detectable (see **Figure 2**).

^5^ GFP fluorescence was monitored in *gfp*-transgenic *N. benthamiana* plants after infection with *X. euvesicatoria* strain 85-10Δ*xopQ* expressing dTALE-2 under control of *vir* and *icm/dot* promoter fragments from respective expression constructs. +, GFP fluorescence; -, no GFP fluorescence detectable; +/-, reduced GFP fluorescence.

In addition to chromosomal *vir* genes, *X. euvesicatoria* contains a second *virB* T4S gene cluster with *virB1 – virB11* genes on plasmid pXCV38 (**Figure 1B**). The region adjacent to *virB1* encodes predicted proteins with homology to TraI and TraG, which are involved in DNA transfer by T4S systems (Audette et al., 2007; Waksman, 2019). TraI belongs to the group of relaxases, which cut DNA prior to transfer at the origin of transfer (*oriT*) and remain attached to the DNA during T4S-dependent transport (Waksman, 2019; Costa et al., 2021). The plasmid-encoded Vir proteins from *X. euvesicatoria* share an overall amino acid identity between 32 – 98% with corresponding proteins encoded by the *virB* gene cluster located on plasmid pXAC64 of *X. axonopodis* pv. *citri* strain 306 (**Figure 1**). In contrast, the amino acid identity between chromosomally and plasmid-encoded Vir proteins is significantly lower in both *X. euvesicatoria* and *X. axonopodis* pv. *citri* and ranges between 20 – 50% (**Figure 1**). In both pathogens, VirD4 is encoded as a single copy gene on the chromosome.

**Figure 2:**
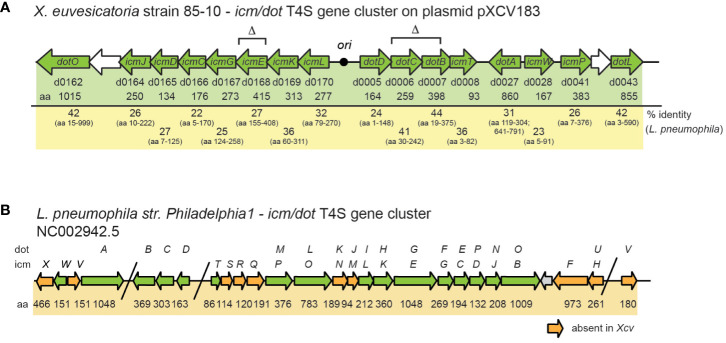
Schematic representation of *icm/dot* T4S gene clusters from *X. euvesicatoria* strain 85-10 and *L. pneumophila* strain *Philadelphia 1.* **(A)** *icm/dot* T4S gene cluster on plasmid pXCV183 from *X. euvesicatoria* strain 85-10. Genes, which are present in both *L. pneumophila* and *X. euvesicatoria*, are represented by green arrows. Gene numbers, the length of the gene products in amino acids (aa) and the amino acid sequence identity with corresponding proteins from *L. pneumophila* are indicated. The black circle refers to the origin of plasmid pXCV183. Δ refers to genes which were deleted in the present study. **(B)** Organization of the *icm/dot* T4S gene cluster from *L. pneumophila* strain *Philadelphia 1.* The nomenclature of genes as *icm* or *dot* genes is given above the arrows. Numbers refer to the size of the corresponding gene products in amino acids (aa). Orange arrows indicate genes, which are absent in *X. euvesicatoria* strain 85-10.

Notably, *X. euvesicatoria* also contains a predicted *icm/dot* T4S gene cluster on plasmid pXCV183 (Thieme et al., 2005) (**Figure 2A**). The corresponding gene products are homologous to major structural components of Icm/Dot T4S systems and share between 22 – 46% amino acid identity with respective Icm/Dot proteins from *L. pneumophila* (**Figure 2A**, **Table S5**). The organization of the 16 putative *icm/dot* genes from *X. euvesicatoria* including *icmL, K, E, G, C, D, J* as well as *dotD, C*, *B*, *O* and *L* is similar to the order of the corresponding genes in *L. pneumophila* (**Figure 2B**). However, genes encoding predicted IcmF, H, N, M, Q, R, S, V and X proteins are missing in *X. euvesicatoria*.

### *vir* and *icm/dot* promoters activate expression of reporter genes *in vitro* and *in planta*

**Figure 3:**
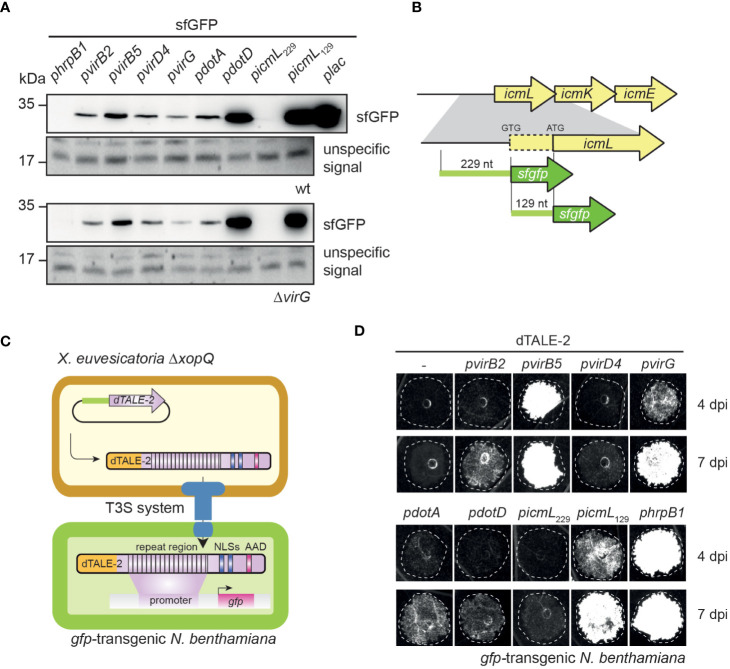
The analysis of promoter-reporter fusions reveals *in vitro* and *in planta* activities of *vir* and *icm/dot* promoters. **(A)** *vir* and *icm/dot* promoters activate expression of the *sfgfp* reporter gene in *X. euvesicatoria.* Strains 85-10 and 85-10Δ*virG* containing *sfgfp* under control of predicted promoters of *virB2, virB5*, *virD4*, *virG*, *dotA*, *dotD* and *icmL* on corresponding expression constructs were grown in NYG medium, and total cell extracts were analyzed by immunoblotting using a GFP-specific antibody. Expression constructs containing *sfgfp* under control of the *hrpB1* promoter, which is inactive in NYG medium, and the *lac* promoter, which is active in *X. euvesciatoria*, were used as negative and positive controls, respectively. The experiment was performed three times with similar results. Unspecific signals detected by the GFP-specific antibody are shown to demonstrate equal loading. Given the high expression level of *gfp* in strain 85-10 containing *gfp* under control of the *plac* promoter, only 0.5 µl of the protein extract were loaded. **(B)** Schematic representation of *icmL* and predicted promoter regions. *icmL* is located upstream of *icmK* and *icmE* in the *icm/dot* T4S gene cluster on plasmid pXCV183 from *X. euvesicatoria* strain 85-10. A putative ATG start codon is located 129 nucleotides (nt) downstream of the annotated GTG translation initiation start site. For the analysis of putative *icmL* promoter regions, 229 nt upstream of the annotated start site and 129 nt upstream of the putative ATG start codon, respectively, were fused to *sfgfp* as indicated. **(C)** Principle of the dTALE-2-based *in vivo* expression and translocation assay. Expression constructs encoding dTALE-2 downstream of the analyzed *vir* or *icm/dot* promoter regions were introduced into *X. euvesicatoria* strain 85-10Δ*xopQ* which is pathogenic on *N. benthamiana* plants (Adlung et al., 2016). For translocation assays, bacteria were infiltrated into *gfp-*transgenic *N. benthamiana* plants which contain a viral vector construct encoding GFP under control of a dTALE-2-responsive promoter. Expression of *dTALE-2* leads to its translocation by the T3S system because dTALE-2 contains an N-terminal secretion and translocation signal (depicted in orange). The central repeat region binds to specific bases in the dTALE-2-responsive promoter. C-terminal nuclear localization signals (NLSs) and an acidic activation domain (AAD) are required for nuclear localization and activation of *in planta* gene expression, respectively. Activation of *gfp* expression by dTALE-2 leads to GFP fluorescence in the infected leaf areas. **(D)** *In vivo* reporter assay for the detection of dTALE-2 translocation. *X. euvesicatoria* strain 85-10Δ*xopQ* with expression constructs containing *dTALE-2* under control of predicted *vir* and *icm/dot* promoters as indicated was infiltrated into leaves of *gfp-*transgenic *N. benthamiana* plants containing *gfp* on a viral vector construct under control of a *dTALE-2-*responsive promoter. *In planta* activation of *vir* and *icm/dot* promoters allowed the synthesis and translocation of dTALE-2 *via* the T3S system, thus inducing GFP fluorescence which was monitored 4 and 7 dpi using a chemiluminescence/fluorescence imager which displays GFP fluorescence in black and white. The experiment was performed three times with similar results.

To investigate whether *vir* and *icm/dot* T4S genes from *X. euvesicatoria* are expressed, we generated reporter constructs containing *sfgfp* (superfolder green fluorescent protein) downstream of predicted promoters of *virB2*, *virB5*, *virD4*, *virG*, *dotA*, *dotD* and *icmL* (see above, **Table 1**). Constructs were transferred into *X. euvesicatoria* strains 85-10 and 85-10Δ*virG*, which lacks the predicted regulatory gene *virG.* The *lac* promoter, which activates gene expression in *X. euvesicatoria*, served as positive control (Szczesny et al., 2010). As negative control, we cloned *sfgfp* downstream of the *hrpB1* promoter, which is inactive in NYG medium (Wengelnik and Bonas, 1996). *hrpB1* is a gene of the T3S gene cluster and is only activated *in planta* or when bacteria are cultivated in special minimal media (Wengelnik and Bonas, 1996). For the analysis of promoter activities, bacteria were cultivated in NYG medium and bacterial cell extracts were analyzed by immunoblotting using a GFP-specific antibody. As expected, sfGFP was detectable in cell extracts of cultures containing *sfgfp* under control of the *lac* but not of the *hrpB1* promoter (**Figure 3A**). All tested *vir* promoters as well as the *dotA* and *dotD* promoters led to sfGFP synthesis. Similar sfGFP levels were obtained when constructs were analyzed in strains 85-10 and 85-10Δ*virG*, suggesting that VirG is dispensable for the activation of gene expression (**Figure 3A**). Notably, the predicted promoter region spanning 229 bp upstream of the annotated start site of *icmL* did not lead to detectable *sfgfp* expression (**Figures 3A, B**).

In addition to the *in vitro* studies, we analyzed a potential *in planta* activity of *vir* and *icm/dot* promoters. For this, we generated expression constructs containing *vir* and *icm/dot* promoters upstream of an artificial *TAL* (transcription activator-like) effector gene (dTALE-2, designer TAL effector-2). dTALE-2 is a derivative of the type III effector protein AvrBs3 from *X. euvesicatoria* which is imported into the plant cell nucleus where it acts as a transcription factor and activates plant gene expression (Boch et al., 2014). TAL effectors bind to defined nucleotide sequences in the promoters of target genes *via* their central region, which consists of a variable number of amino acid repeats and allows the base-specific binding to DNA (Boch et al., 2009; Moscou and Bogdanove, 2009). dTALE-2 is used as a reporter for T3S-dependent protein translocation because it binds to a dTALE-2-responsive promoter which controls the expression of *gfp* on a stably integrated viral vector construct in transgenic *Nicotiana benthamiana* plants (Werner et al., 2011; Scheibner et al., 2016). *vir* and *icm/dot* promoter-driven expression in *X. euvesicatoria*, which leads to the translocation of dTALE-2 by the T3S system, therefore activates *gfp* expression in transgenic *N. benthamiana* plants (**Figures 3C, D**). For these assays, expression constructs encoding dTALE-2 under control of *vir* or *icm/dot* promoters were transferred into *X. euvesicatoria* strain 85-10Δ*xopQ* which lacks the T3E XopQ and induces disease symptoms in *N. benthamiana* plants (Adlung et al., 2016). Inoculation of *gfp-*transgenic *N. benthamiana* plants with *xopQ* deletion mutants containing *dTALE-2* expression constructs led to GFP fluorescence 4 dpi when *dTALE-2* was expressed under control of the *virB5*, *virG* or *hrpB1* promoter (**Figure 3D**). Expression of *dTALE-2* under control of the *virB2*, *dotA* and *dotD* promoters led to weaker fluorescence which was detectable 7 dpi (**Figure 3D**). No GFP fluorescence was observed when *dTALE-2* was expressed under control of the *virD4* or the *icmL* promoter (**Figure 3D**). The lack of detectable GFP fluorescence after *virD4* promoter-driven expression of *dTALE-2* is in contrast to the *in vitro* activity of the *virD4* promoter and suggests that *virD4* is not or only weakly expressed *in planta* (**Figures 3A, D**). In case of the predicted *icmL* promoter, we assume that the region 229 nucleotides upstream of the annotated GTG start codon of *icmL* does not activate *in vitro* or *in planta* reporter gene expression. Interestingly, DNA sequence analyses revealed the presence of an additional putative ATG translation initiation codon 129 nucleotides downstream of the predicted GTG start codon of *icmL* (**Figure 3B**). To investigate whether the *icmL* promoter is located between the annotated GTG and the alternative ATG start codon, we generated an additional expression construct containing *dTALE-2* under control of a 129-bp DNA fragment upstream of the ATG codon. This led to detectable *in vitro* and *in planta* reporter gene expression, indicative of a promoter downstream of the annotated GTG translation start site (**Figures 3A, D**). In agreement with this finding, a promoter and a *vir* box was predicted in the region spanning 129 bp downstream of the annotated GTG start codon (**Table 1** and **Table S4**).

### T4S genes are dispensable for virulence of *X. euvesicatoria*

**Figure 4:**
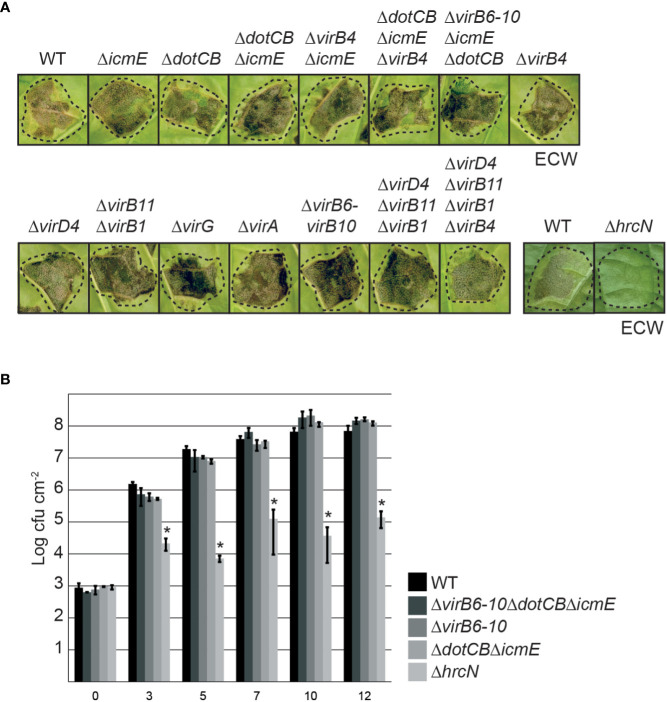
The putative Vir- and Icm/Dot-like T4S systems do not significantly contribute to virulence of *X. euvesicatoria.* **(A)** Infection studies with *X. euvesicatoria* wild-type, *hrcN* and T4S mutant derivatives. Strain 85-10 (wt) and deletion mutant derivatives thereof lacking the T3S ATPase gene *hrcN* or single or multiple *vir* and *icm/dot* genes as indicated were infiltrated at a density of 10^8^ cfu ml^-1^ into leaves of susceptible ECW pepper plants. Disease symptoms were photographed 7 dpi. Dashed lines indicate the inoculated leaf areas. The experiment was performed three times with similar results. **(B)** *In planta* growth of wild-type and T4S mutant derivatives of *X. euvesicatoria.* Strain 85-10 (wt) and deletion mutant derivatives thereof lacking single or multiple *vir* and *icm/dot* genes as indicated were infiltrated into leaves of three susceptible ECW pepper plants. Bacterial *in planta* growth was monitored over a period of 12 days. The T3S-deficient and non-pathogenic strain 85-10Δ*hrcN*, which lacks the ATPase of the T3S system, was analyzed as control. Mean values of cfu/cm^2^ and standard deviations from one experiment are depicted in the diagram. The experiment was performed three times with similar results. According to a one-tailed paired t-test, the difference in growth of *X. euvesicatoria* wt and *hrcN* deletion mutant strains is significant (with a p value of 0.024; indicated by asterisks). Growth of T4S mutants is not significantly different from that of the wild-type strain (p values of 0.075 for strain 85-10Δ*virB6-10ΔdotCBΔicmE*, 0.121 for strain 85-10Δ*virB6-10* and 0.134 for strain 85-10Δ*dotCBΔicmE*).

To analyze a potential virulence function of T4S genes in *X. euvesicatoria* strain 85-10, we deleted single and multiple *vir* and *icm/dot* genes encoding predicted essential components of the T4S systems such as ATPases (VirD4, VirB4, VirB11 and DotB), the predicted structural components VirB6, VirB8, VirB9, VirB10, DotC and IcmE, and the putative regulators VirA and VirG (**Figures 1**, **2**). For infection experiments, the wild-type *X. euvesicatoria* strain 85-10 and corresponding deletion mutants were infiltrated into leaves of susceptible ECW pepper plants and disease symptoms were inspected over a period of five to eight days. As expected, the wild-type strain 85-10 induced disease symptoms in form of water-soaked lesions that became necrotic five to seven dpi (**Figure 4A**). No reduction in symptom formation was detectable after infiltration of mutant strains deleted in single or multiple *vir* or *icm/dot* genes (**Figure 4A**). To investigate a potential redundant virulence function of both T4S systems, we also combined deletions in *vir* and *icm/dot* genes, thus generating strains 85-10Δ*virB4*Δ*icmE*, 85-10Δ*dotCB*Δ*icmE*Δ*virB4* and 85-10Δ*virB6*-*10ΔicmEΔdotCB*. However, no significant reduction in disease symptoms was observed (**Figure 4A**).

In addition to phenotypic analyses, we performed *in planta* growth curves with strains 85-10, 85-10Δ*virB6-10*, 85-10Δ*dotCB*Δ*icmE* and 85-10Δ*virB6-10*Δ*dotCB*Δ*icmE.* For this, bacteria were infiltrated into leaves of susceptible ECW pepper plants, and bacterial growth was analyzed over a period of twelve days. As control, we included the non-pathogenic strain 85-10Δ*hrcN* which lacks the ATPase HrcN of the T3S system (Lorenz and Büttner, 2009). The wild-type strain 85-10 multiplied up to 10^8^ cfu ml^-1^ whereas a significant reduction in bacterial growth was observed for strain 85-10Δ*hrcN* as expected (**Figure 4B**). T4S mutant strains displayed a wild-type growth rate, suggesting that *vir* and *icm/dot* genes do not significantly contribute to bacterial multiplication *in planta* (**Figure 4B**).

### Cocultivation with *X. euvesicatoria* does not significantly affect *in vitro* growth of *E. coli*

**Figure 5:**
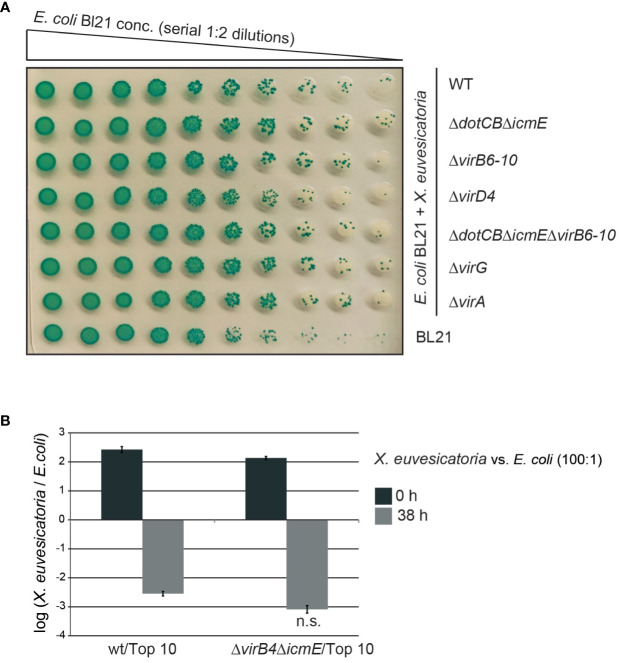
*In vitro* growth *of E. coli* is unaffected by *X. euvesicatoria.* **(A)** Cocultivation with *X. euvesicatoria* strains does not affect *in vitro* growth of *E. coli*. Serial dilutions of *E. coli* strain BL21, which naturally expresses the β-galactosidase, were incubated with *X. euvesicatoria* strain 85-10 (wt) or deletion mutant derivatives thereof lacking single or multiple *vir* and *icm/dot* genes as indicated. *X. euvesicatoria* strains were incubated at initial concentrations of 5 × 10^8^ CFU/ml with *E. coli* BL21 at a concentration of 5 × 10^6^ CFU/ml or serial 1:2 dilutions on LB agar plates containing X-gal and IPTG overnight. One representative image is shown. The experiment was performed three times with similar results. **(B)** Ratio of *X. euvesicatoria* and *E. coli* cells after cocultivation. *X. euvesicatoria* strain 85-10 (wt) or 85-10Δ*icmEΔvirB4* and *E. coli* strain Top 10 were mixed at a ratio of 100: 1, spotted on an LB agar plate and serial dilutions were plated after 0 and 38 hours on NYG medium with rifampicin for *X. euvesicatoria* strains and on LB medium with streptomycin for growth of *E. coli* to count bacterial colonies. Mean values and standard deviations of the ratio of *X. euvesicatoria* and *E. coli* colonies from one representative experiment are shown. According to a t-test, the difference in growth of *E. coli* in the presence of the *X. euvesicatoria* wt or the T4S mutant is not significant (n.s.; p value of 0.2487). The experiment was performed three times with similar results.

The chromosomally encoded VirB/VirD4 T4S system from *X. axonopodis* pv. *citri* and the corresponding T4S system from the related pathogen *S. maltophilia* were previously shown to deliver toxins into Gram-negative bacteria such as *E. coli*, thus resulting in cell lysis (Souza et al., 2015; Bayer-Santos et al., 2019). To investigate a similar toxic effect of *X. euvesicatoria* on *E. coli*, we incubated *X. euvesicatoria* strain 85-10 and deletion mutants thereof lacking single or multiple *vir* or *icm/dot* genes in the presence of decreasing concentrations of *E. coli* strain BL21. Co-cultures were spotted on LB agar plates containing X-Gal which is a chromogenic artificial substrate of the β-galactosidase from *E. coli* and is converted into a blue pigment. The formation of blue colonies by *E. coli* indicates bacterial multiplication and was unaffected upon cocultivation with *X. euvesicatoria* (**Figure 5A**).

In additional experiments, we incubated *X. euvesicatoria* wild-type and T4S mutant strains with *E. coli* strain Top10 in a ratio of 100:1 on solid LB medium and plated the bacteria 0 and 38 hours later for quantitative analysis. The *X. euvesicatoria/E. coli* ratio decreased 38 h after incubation, indicating a faster growth rate of *E. coli* compared to *X. euvesicatoria* (**Figure 5B**). However, no significant difference with *X. euvesicatoria* wild-type and T4S mutant strains was observed, suggesting that the T4S systems from *X. euvesicatoria* do not exert a negative effect on the growth of *E. coli* upon cocultivation under the tested conditions (**Figure 5B**). This is in contrast to the VirB/VirD4 T4S system from *X. axonopodis* pv. *citri* which exerts a toxic effect on *E. coli* (Souza et al., 2015).

### The VirB/VirD4 T4S system from *X. euvesicatoria* mediates horizontal gene transfer

**Figure 6:**
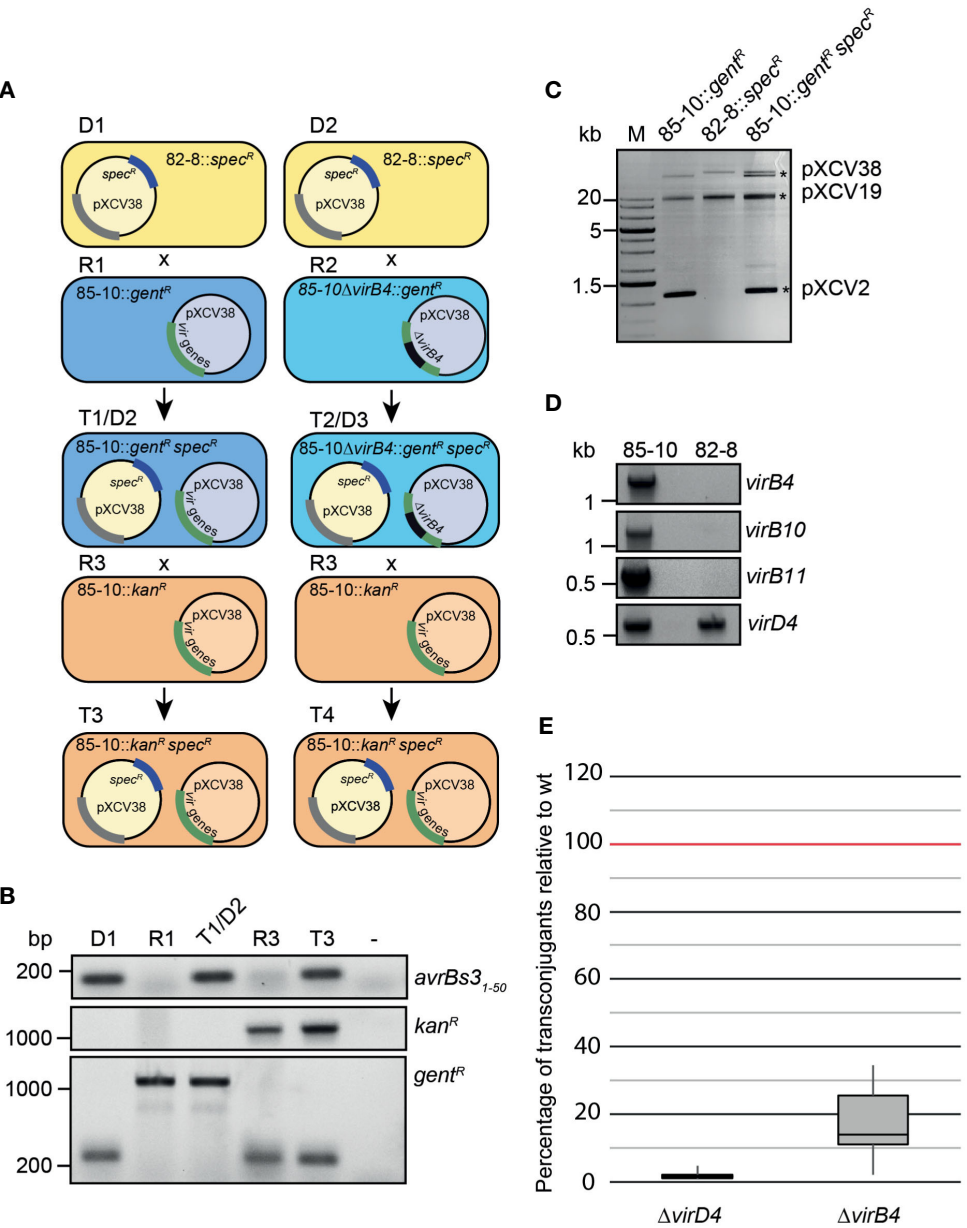
Plasmid transfer by *X. euvesicatoria* strain 85-10 is controlled by VirD4. **(A)** Schematic overview on conjugation experiments with *X. euvesicatoria* strains. Plasmid pXCV38 from strain 82-8 (donor 1, D1) containing a spectinomycin resistance gene (pXCV38_82-8_::*spec^R^*) was transferred by conjugation to strain 85-10::*gent^R^* (recipient 1, R1) which contains the *vir* gene cluster on plasmid pXCV38. A grey bar indicates that the presence of a similar cluster on plasmid pXCV38_82-8_::*spec^R^* is still unknown. The resulting transconjugant T1 (referred to as 85-10::*gent^R^ spec^R^*) was gentamicin- and spectinomycin-resistant and contained both plasmids. The numbers of transconjugants from three independent experiments resulting from the transfer of plasmid pXCV38_82-8_::*spec^R^* is given in **Table S6**. Similarly, pXCV38_82-8_::*spec^R^* was transferred to a derivative of strain 85-10::*gent^R^* with a deletion in *virB4* (85-10Δ*virB4*::*gent^R^*; recipient 2, R2), resulting in transconjugants T2. T1 and T2 served as donor strains (D2 and D3) in a second conjugation experiment using strain 85-10::*kan^R^*, which contains a kanamycin resistance gene, as recipient (R3). The resulting transconjugants (T3 and T4) grew on medium with kanamycin and spectinomycin and contained plasmid pXCV38_82-8_::*spec^R^*. In addition to the *virB4* deletion mutant, we used *virD4* mutant strains for the conjugation experiments (see panel E). *virD4* is located on the chromosome and absent from plasmid pXCV38. **(B)** Confirmation of plasmid transfer by PCR analysis of transconjugants. The first 50 codons of *avrBs3*, the kanamycin and the gentamicin resistance gene were amplified by PCR using donor, recipient and transconjugant strains as depicted in **(A)** as templates. Amplicons were analyzed by agarose gel electrophoresis and visualized by an UV imager. Primers used to amplify the gentamicin resistance gene annealed to the chromosomal *hpaFG* region, thus leading to a smaller PCR product in the absence of the gentamicin resistance gene. **(C)** Analysis of isolated plasmids from *X. euvesicatoria* by agarose gel electrophoresis. Plasmids were isolated from *X. euvesicatoria* strains 85-10::*gent^R^*, 82-8 pXCV38_82-8_::*spec^R^* (82-8::*spec^R^*) and 85-10::*gent^R^* pXCV38_82-8_::*spec^R^* (85-10::*gent^R^ spec^R^*) and separated by agarose gel electrophoresis. Asterisks indicate the expected sizes of plasmids pXCV2 (1852 bp), pXCV19 (19146 bp) and pXCV38 (38116 bp) from *X. euvesicatoria* strain 85-10. Plasmid pXCV183 (182572 bp), which is also present in strain 85-10, presumably did not migrate into the agarose gel. **(D)** PCR amplification of *vir* genes from *X. euvesicatoria* strains 85-10 and 82-8. *vir* genes were amplified by PCR using specific primers for *virB4*, *virB10, virB11* and *virD4* from strain 85-10 and genomic DNA of strains 85-10 and 82-8 as template. PCR products were analyzed by agarose gel electrophoresis and visualized by an UV imager. **(E)** VirD4 and VirB4 are required for efficient plasmid transfer. Donor and recipient were mixed at a ratio of 1:1 and incubated overnight on a cellulose nitrate filter placed on solid NYG medium and covered with 1% water agar. Serial dilutions were plated on selective plates to count the transconjugants. The highest numbers of transconjugants were obtained when the T4S wild-type strain 85-10::*gent^R^* containing pXCV38_82-8_::*spec^R^* was used as donor and were set to 100%. Reduced numbers of transconjugants obtained with strains 85-10Δ*virD4* and 85-10Δ*virB4* both containing pXCV38_82-8_::*spec^R^* are depicted in a boxplot diagram. Values and standard deviations represent the percentage of transconjugants from six experiments with strain 85-10Δ*virD4* and from ten experiments with strain 85-10Δ*virB4*.

The association of *traI* and *traG* genes with the plasmid-localized *vir* gene cluster from *X. euvesicatoria* prompted us to investigate the role of the predicted VirB/VirD4 T4S system in plasmid transfer. It was previously reported that *X. euvesicatoria* and *X. citri* pv. *citri* transfer plasmids and chromosomal DNA into other *Xanthomonas* strains (Basim et al., 1999; El Yacoubi et al., 2007). In the present study, we analyzed the transfer of a derivative of plasmid pXCV38 (hereafter referred to as pXCV38_82-8_:*:spec^R^*) which originates from strain 82-8 and contains a spectinomycin resistance gene inserted into the *avrBs3* T3E gene (Bonas et al., 1989). Donor and recipient strains with plasmid pXCV38_82-8_:*:spec^R^* were, therefore, selected on medium containing spectinomycin. For conjugation experiments, strain 82-8 with plasmid pXCV38_82-8_:*:spec^R^* was incubated with a derivative of strain 85-10 containing a gentamicin resistance gene inserted into the genome in the *hpaFG* region adjacent to the *hrp* gene cluster. The *hpaFG* region is dispensable for virulence and serves as a landing platform for gene insertion (Lorenz et al., 2012). Cocultivation of strains 82-8 pXCV38_82-8_:*:spec^R^* and 85-10::*gent^R^* resulted in transconjugants which were gentamicin- and spectinomycin-resistant, suggesting that pXCV38_82-8_:*:spec^R^* had been transferred from strain 82-8 into strain 85-10::*gent^R^* (**Figure 6A** and **Table S6**). The presence of pXCV38_82-8_:*:spec^R^* in the recipient strains was confirmed by PCR amplification of the 5’ region of *avrBs3* and by agarose gel electrophoresis of isolated plasmids (**Figures 6B and C**). pXCV38_82-8_:*:spec^R^* from strain 82-8, which has not yet been sequenced, is larger than pXCV38 from strain 85-10. Both plasmids could, therefore, be separated by agarose gel electrophoresis and were present in the resulting transconjugants (**Figure 6C**). Strain 82-8 thus likely contains a functional T4S system which acts as a conjugation machine and mediates transfer of plasmid pXCV38_82-8_:*:spec^R^*. To investigate whether strain 82-8 contains *vir* genes, we performed PCR analysis using primers specific for *vir* genes from strain 85-10. Notably, we did not amplify *virB4*, *virB10* and *virB11* from strain 82-8, suggesting that the primers did not efficiently bind to the template DNA (**Figure 6D**). In contrast, the chromosomal *virD4* gene was amplified from strains 85-10 and 82-8 (**Figure 6D**). We assume that strain 82-8 contains a functional T4S system because it mediates transfer of plasmid pXCV38_82-8_:*:spec^R^* (see above).

To investigate a possible contribution of the T4S systems to plasmid transfer in strain 85-10, pXCV38_82-8_:*:spec^R^* was transferred to derivatives of strain 85-10::*gent^R^* deleted in *virD4 or virB4* (**Figure 6A**). The resulting T4S wild-type and mutant derivatives of strain 85-10::*gent^R^* containing pXCV38_82-8_:*:spec^R^* served as donor strains in additional conjugation experiments, using a kanamycin-resistant derivative of strain 85-10 (85-10::*kan^R^*) as recipient (**Figure 6A**). Transconjugants grew on medium containing kanamycin and spectinomycin but were sensitive to gentamicin, indicating a transfer of pXCV38_82-8_:*:spec^R^* from strain 85-10::*gent^R^* to strain 85-10::*kan^R^* (data not shown). PCR analyses confirmed the presence of the kanamycin resistance gene and the 5’ region of *avrBs3* in the transconjugants (**Figure 6B**). pXCV38_82-8_:*:spec^R^* was most efficiently transferred by strain 85-10::*gent^R^*, whereas significantly less transformants were obtained when strain 85-10Δ*virD4*::*gent^R^* was used as donor strain (**Figure 6E**). Residual plasmid transfer was observed with strain 85-10Δ*virB4*::*gent^R^*, resulting in approximately 20% of the transconjugants obtained with the wild-type strain (**Figure 6E**). It is possible that the loss of VirB4 was partially compensated by functionally redundant proteins encoded in the genome of the donor strain or on plasmid pXCV38_82-8_:*:spec^R^*, which was used to analyze the plasmid transfer. However, given the severe reduction in plasmid transfer by the *virD4* mutant, we assume that the VirB/VirD4 T4S system acts as a conjugation machine and mediates horizontal gene transfer.

### Bacterial two-hybrid studies revealed homo- and heterooligomerization of Vir and Icm/Dot proteins

Next, we investigated whether VirB/VirD4 and Icm/Dot proteins from *X. euvesicatoria* assemble into oligomeric complexes as would be expected from their predicted functions as components of T4S systems. For protein-protein interaction studies, we used the bacterial adenylate cyclase-based two-hybrid (BACTH) system which detects the interaction of proteins fused to the T18 or the T25 subdomain of the catalytic region of the adenylate cyclase (Cya). Interaction of T18 and T25 fusions leads to the reconstitution of Cya and thus to the production of cAMP. This activates the expression of the *lac* operon in *E. coli* reporter strains lacking the native *cya* gene (Karimova et al., 1998; Battesti and Bouveret, 2012). LacZ activity results in blue colonies, indicative of an interaction between both fusion proteins.

**Figure 7:**
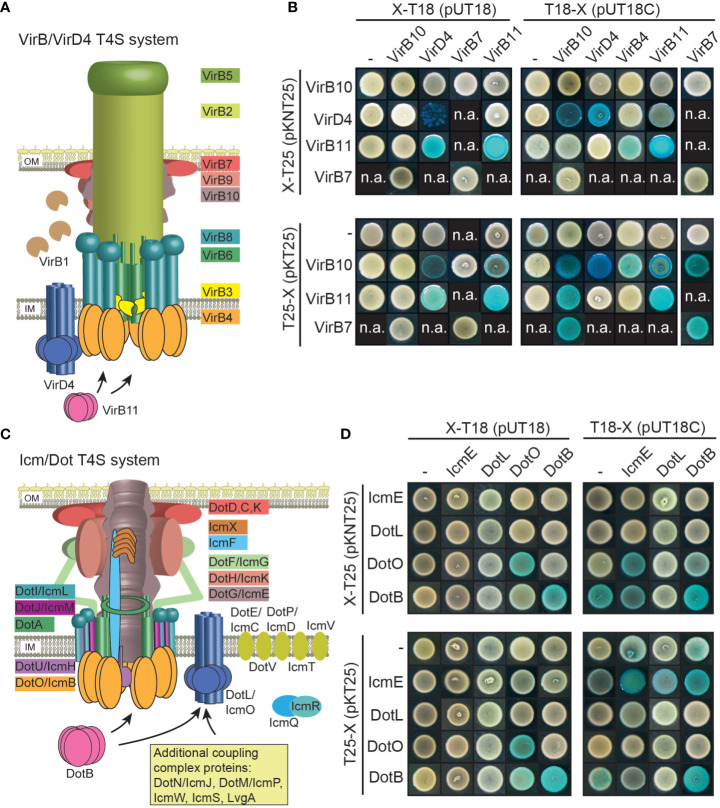
Vir and Icm/Dot proteins from *X. euvesicatoria* assemble into oligomeric complexes. **(A)** Schematic representation of the VirB/VirD4 T4S system. The model was adapted according to Mace et al., 2022. IM, inner membrane; OM, outer membrane. **(B)** Results of BACTH assays with Vir proteins from *X. euvesicatoria.* T18 and T25 fusions of the predicted structural components VirB7 and VirB10 as well as of the putative ATPases VirD4, VirB4 and VirB11 were analyzed in *E*. *coli* BTH101 cells as indicated and bacterial cultures were grown on indicator plates containing X-gal and IPTG. As control, fusion proteins were tested against the T18 or T25 domain alone as indicated. All proteins were stably synthesized as was shown by immunoblot analysis of bacterial cell extracts, using a FLAG epitope-specific antibody (see **Figure S1**). n.a., not analyzed. Interaction studies were performed at least three times with similar results. One representative colony is shown. Results are summarized in **Table 2**. **(C)** Schematic representation of the Icm/Dot-type T4S system. The model was adapted according to previous publications (Christie et al., 2017; Ghosal et al., 2019; Gomez-Valero et al., 2019; Sheedlo et al., 2022). IM, inner membrane; OM, outer membrane. **(D)** BACTH assays with Icm/Dot proteins from *X. euvesicatoria.* T18 and T25 fusions of the predicted structural component IcmE and the putative ATPases DotL, DotO and DotB were analyzed as described in **(B)**. All proteins were stably synthesized as was shown by immunoblot analysis of bacterial cell extracts, using a FLAG epitope-specific antibody (see **Figure S1**). n.a., not analyzed. Interaction studies were performed at least three times with similar results. Results are summarized in **Table 2**.

We performed BACTH assays with T18 and T25 fusions of the predicted ATPases VirD4, VirB4 and VirB11 as well as of the predicted core components of the VirB/VirD4 T4S system VirB7 and VirB10 (**Figure 7A**). Corresponding expression constructs were cotransformed into the *E. coli* BTH101 reporter strain and transformants were grown on plates containing X-Gal. We detected homo- and heterooligomerizations of the putative ATPases VirD4 and VirB11 as well as interactions between all tested putative ATPases with VirB10 (**Figure 7B** and **Table 2**). Furthermore, VirB7 interacted with itself and with VirB10 (**Figure 7B** and **Table 2**). The interactions were specific because the analyzed T4S system components did not interact with the T25 and T18 domains alone (**Figure 7B**). Immunoblot analysis of bacterial cell extracts showed that most tested T25 and T18 fusion proteins were stably synthesized (**Figure S1**). T25-VirB4 was not detectable and was, therefore, not included in the analyses.

**Table 2:** Summary of the results of BACTH assays with components of VirB/VirD4 and Icm/Dot T4S substrates.^1^.

	VirB10	VirD4	VirB4	VirB11	VirB7	TraG	IcmE	DotL	DotO	DotB
VirB10	+	+	+	+	+	+	n.a.	n.a.	n.a.	n.a.
VirD4	+	+	-	+	n.a.	n.a.	n.a.	n.a.	n.a.	n.a.
VirB4	+	-	n.a.	+	n.a.	n.a.	n.a.	n.a.	n.a.	n.a.
VirB11	+/-	+	+	+	n.a.	+	n.a.	n.a.	n.a.	n.a.
VirB7	+	n.a.	n.a.	n.a.	+	n.a.	n.a.	n.a.	n.a.	n.a.
TraG	+	n.a.	n.a.	+	n.a.	+	n.a.	n.a.	n.a.	n.a.
IcmE	n.a.	n.a.	n.a.	n.a.	n.a.	n.a.	+	+	+	+/-
DotL	n.a.	n.a.	n.a.	n.a.	n.a.	n.a.	-	-	-	-
DotO	n.a.	n.a.	n.a.	n.a.	n.a.	n.a.	+	-	+	-
DotB	n.a.	n.a.	n.a.	n.a.	n.a.	n.a.	-	-	-	+

^1^ +, interaction; +/-, reduced interaction; -, no interaction detectable; n.a., not analyzed.Yellow and green boxes refer to interactions between components of the VirB/VirD4 and the Icm/Dot T4S system, respectively. Interactions, which were not analysed, are listed in grey boxes.

In addition to components of the predicted VirB/VirD4 T4S system, we performed BACTH assays with Icm/Dot proteins including the predicted ATPases DotO and DotB, which correspond to VirB4 and VirB11, respectively (Sheedlo et al., 2022) (**Figure 7C**). We also performed BACTH assays with the predicted coupling protein DotL as well as the putative major structural component IcmE which is also referred to as DotG and corresponds to VirB10. When analyzed as T25 and T18 fusions, DotO and DotB interacted with themselves as is expected for hexameric ATPases (**Figure 7D** and **Table 2**). Furthermore, a weak interaction between T18-IcmE and DotO-T25 was detected and IcmE interacted with itself (**Figure 7D** and **Table 2**). No self-interaction was observed for the predicted coupling protein DotL (**Figure 7D**). Yet, it cannot be excluded that N- or C-terminal T25 or T18 fusion partners interfere with the self-interaction of DotL. All tested fusion proteins were synthesized as was revealed by immunoblot analysis (**Figure S1**). Taken together, we conclude from the results of the BACTH assays that the predicted ATPases of both T4S systems self-interact as expected. Furthermore, VirD4, VirB4 and VirB11 interact with VirB10, which is the putative major structural component of the VirB/VirD4 T4S system and also associates with VirB7 (**Table 2**). Our results thus suggest that Vir and Icm/Dot proteins are involved in the assembly of heterooligomeric protein complexes, likely corresponding to T4S systems.

### ATPases of T4S systems interact with potential T4S substrates

**Figure 8:**
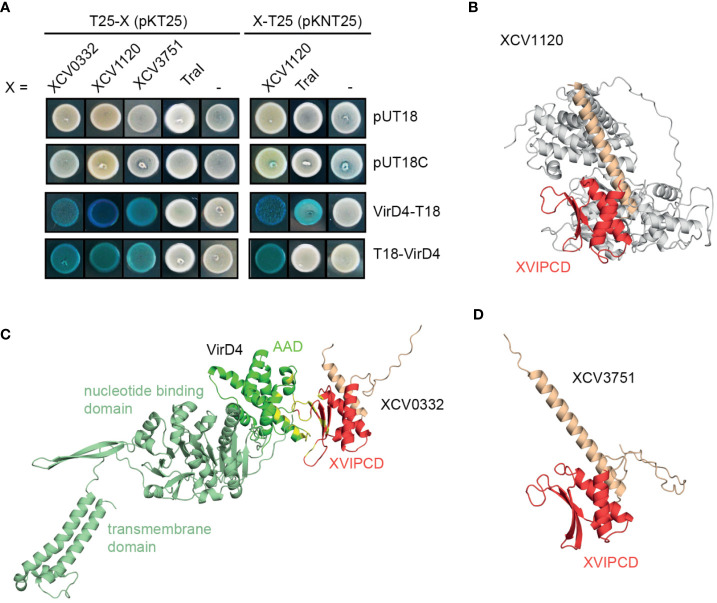
Candidate T4S substrates from *X. euvesicatoria* interact with VirD4 and contain a predicted ααβββ motif in the C-terminal XVIPCD. **(A)** Candidate T4S substrates from *X. euvesicatoria* interact with VirD4 in BACTH-based interaction studies. The interaction of T18 and T25 fusions of the candidate T4S substrates XCV0332, XCV1120, XCV3571 and TraI with VirD4 was analyzed in *E*. *coli* BTH101 cells as indicated and bacterial cultures were grown on indicator plates containing X-gal and IPTG. As control, fusion proteins were tested against the T18 or T25 domain alone as indicated. All tested proteins were stably synthesized as was shown by immunoblot analysis of bacterial cell extracts, using a FLAG epitope-specific antibody (see **Figure S1**). Interaction studies were performed at least three times with similar results. One representative colony is shown. **(B)** Structure prediction of VirD4 and in complex with the T4S substrate XCV0332 from *X. euvesicatoria.* Structures were predicted using the AlphaFold 2 algorithm and visualized using PyMOL (https://pymol.org). The AAD from VirD4 is shown in dark green, the ααβββ motif in the XVIPCDs of XCV0332 is shown in red. Residues predicted to be involved in the interaction between the AAD from VirD4 and the XVIPCD of XCV0332 are shown in yellow. **(C)** Structure prediction of XCV1120 from *X. euvesicatoria.* using the AlphaFold 2 algorithm. The ααβββ motif in the XVIPCDs is shown in red. **(D)** Structure prediction of XCV3751 from *X. euvesicatoria.* using the AlphaFold 2 algorithm. The ααβββ motif in the XVIPCDs is shown in red.

To identify possible substrates of the VirB/VirD4 T4S system from *X. euvesicatoria*, we searched for homologs of known type IV effectors from *X. axonopodis* pv. *citri* which are referred to as X-Tfes and were previously identified due to their interaction with the T4CP VirD4 (Alegria et al., 2005). In the present study, we focused on the X-Tfe homologs XCV0332, XCV1120 and XCV3571, which contain C-terminal motifs of XVIPCDs such as blocks of conserved amino acid sequences (GLxRIDHV and FAVQ GxxDPAHxRAHV; x refers to any residue) and a glutamine-rich C-terminal tail (Alegria et al., 2005) (**Tables 3** and **S7**). Given that the XVIPCD provides the binding site for the AAD of VirD4 from *X. axonopodis* pv. *citri* (Alegria et al., 2005; Oka et al., 2022), we investigated possible interactions of the identified T4S candidate substrates with VirD4 from *X. euvesicatoria.* BACTH assays revealed interactions of T25 fusions of XCV3751, XCV1120 and XCV0332 with T18 fusions of VirD4 (**Figure 8A** and **Table 2**). The interactions were specific for VirD4, because the analyzed VirD4 interaction partners did not interact with the T18 domain alone (**Figure 8A**). Immunoblot analyses showed that most fusion proteins were stably synthesized (**Figure S1**). Exceptions were XCV0332-T25 and XCV3751-T25, which were, therefore, not included in the analyses (**Figure S1**). It was previously reported that the N-terminal region of the XVIPCD from X-Tfes adopts an ααβββ fold which interacts with the AAD of VirD4 (Oka et al., 2022). Interestingly, *in silico* modelling using the AlphaFold 2 algorithm (Jumper et al., 2021) revealed a similar fold of XVIPCDs in XCV0332, XCV1120 and XCV3751 which might associate with the AAD of VirD4 from *X. euvesicatoria* (**Figures 8B-D**). As additional candidate T4S substrate, we analyzed TraI which is a predicted relaxase and likely secreted by the VirB/VirD4 T4S system. TraI lacks an XVIPCD but also interacted with VirD4 when analyzed as T25 fusion (**Figure 8A** and **Table 2**).

**Table 3:** Characteristics of candidate T4S substrates from *X. euvesicatoria* strain 85-10.

Protein (size in aa)	Size in aa^1^	Predicted function	Homologs from *Xac* ^2^	aa identity (%)^3^	XVIPCD^4^		
					GLxRIDHV	FAVQGxxDPAHxRAHV	Q stretch
XCV0332	130	Hypothetical protein	XAC0323^1^	95	–	+	–
XCV1120	160	Hypothetical protein	XAC3266^1^	75	(+)	–	+
XCV3751	630	Peptidoglycan-binding domain	XAC3634^1^	97	+	+	+
TraI	991	Relaxase	TraI	96	–	–	+

^1^ aa, amino acids.

^2^ Proteins from *Xac* (*X. axonopodis* pv. *citri* strain 306) are predicted X-Tfes and were previously identified by yeast two-hybrid approaches using VirD4 as bait (Alegria et al., 2005).

^3^ In case of XCV3751, the identity XAC3634 from *X. axonopodis* pv. *citri* is restricted to amino acids 325 – 630 of XCV3751.

^4^ The XVIPCD contains blocks of conserved amino acid sequences including GLxRIDHV, FAVQGxxDPAHxRAHV and a glutamine-rich C-terminal tail (Alegria et al., 2005). x refers to any residue.

Given the results of our interaction and mutant studies, we assume that VirD4, which is encoded on the chromosome, acts as coupling protein for the VirB-like T4S system encoded on plasmid pXCV38. It can, however, not be excluded that substrate recognition is mediated by TraG which is encoded next to the *virB* T4S gene cluster on plasmid pXCV38 and shares 23% amino acid identity with VirD4 (**Figure 1** and **Figure S2**). Since TraG-like proteins were shown to contribute to T4S (Schröder et al., 2002; Khara et al., 2021; Wang et al., 2022), we analyzed a possible interaction of TraG with components and putative T4S substrates of the VirB-like T4S system. When analyzed by BACTH assays, TraG interacted with itself, with the predicted ATPase VirB11 and the structural component VirB10 but not with the potential T4S substrate TraI (**Figure S2**). This is in contrast to VirD4 and suggests that VirD4 rather than TraG is involved in substrate recruitment.

### TraI and XCV0332 are secreted by the T4S systems from *X. euvesicatoria*

**Figure 9:**
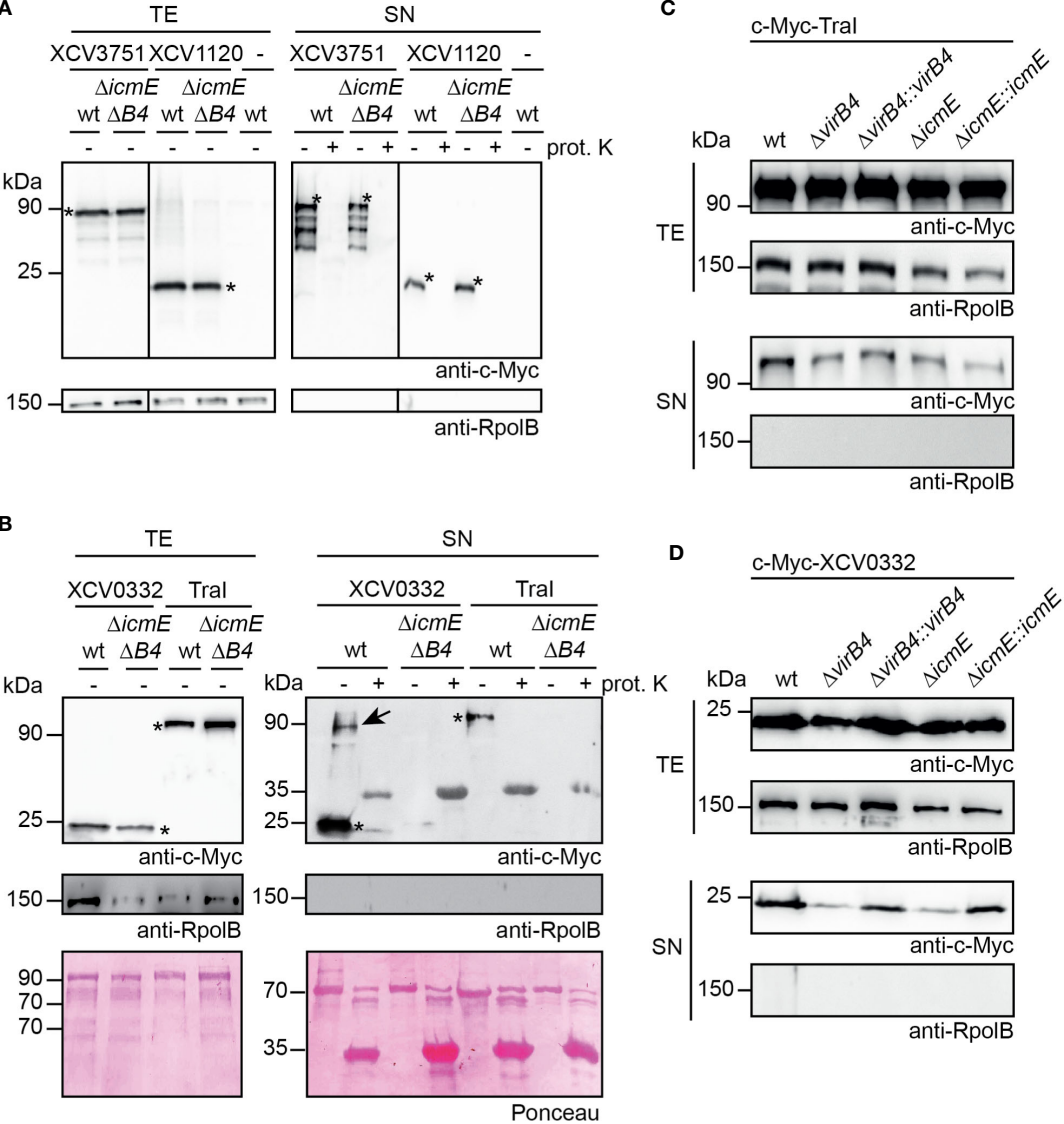
T4S systems from *X. euvesicatoria* secrete XCV0332 and TraI. **(A)** XCV3751 and XCV1120 are secreted independently of the T4S systems. *X. euvesicatoria* strains 85-10 (wt) and 85-10Δ*icmEΔvirB4* (Δ*icmEΔB4*) with or without (-) expression constructs encoding c-Myc-XCV3751 and c-Myc-XCV1120 as indicated were incubated in NYG medium containing BSA. Total cell extracts (TE) and culture supernatants (SN) were analyzed by immunoblotting using a c-Myc epitope-specific antibody. To investigate whether proteins were secreted *via* OM vesicles, SN were incubated in the presence (+) or absence (-) of proteinase K at a final concentration of 0.1 mg/µl. Blots were reprobed with an antibody specific for the RNA polymerase B (RpolB) to analyze whether the presence of proteins in the culture supernatant was caused by cell lysis. Asterisks indicate signals corresponding to the expected sizes of c-Myc-XCV3751 or c-Myc-XCV1120, additional signals presumably represent degradation products. **(B)** *In vitro* secretion assays with XCV0332 and TraI. *X. euvesicatoria* strains 85-10 (wt) and 85-10Δ*icmEΔvirB4* with expression constructs encoding c-Myc-XCV0332 or c-Myc-TraI as indicated were incubated in NYG medium containing BSA. TE and SN were analyzed by Ponceau staining and by immunoblotting using c-Myc- and RpolB-specific antibodies. SN were incubated in the presence (+) or absence (-) of proteinase K as described in **(A)**. Asterisks indicate signals corresponding to the expected sizes of c-Myc-XCV0332 or c-Myc-TraI. The signal at a size of approximately 35 kDa results from unspecific binding of the antibodies to proteinase K. The signal at 90 kDa (indicated by an arrow) detected in the culture supernatant of strain 85-10 containing c-Myc-XCV0332 presumably corresponds to a XCV0332-containing protein complex. **(C)** The predicted relaxase TraI is secreted in *icmE* and *virB4* mutants. For *in vitro* secretion assays, c-Myc-TraI was ectopically expressed in strain 85-10 (wt) and deletion mutant derivatives thereof lacking *virB4* or *icmE* as indicated. For complementation studies, *virB4* and *icmE* were reinserted into the genome of the corresponding deletion mutants (Δ*virB4::virB4* and Δ*icmE::icmE*). Bacteria were incubated in NYG medium in the presence of BSA, and TE and SN were analyzed by immunoblotting, using c-Myc epitope- and RpolB-specific antibodies. **(D)** VirB4 and IcmE contribute to the secretion of XCV0332. *X. euvesicatoria* strains as described in **(C)** and containing c-Myc-XCV0332 were incubated in NYG medium containing BSA, and TE and SN were analyzed by immunoblotting, using c-Myc epitope- and RpolB-specific antibodies. All secretion assays were performed at least three times with similar results.

To analyze a possible *in vitro* secretion of candidate T4S substrates from *X. euvesicatoria*, we generated expression constructs encoding XCV3751, XCV1120, XCV0332 and TraI fused to an N-terminal c-Myc epitope under control of the *lac* promoter. We assume that an N-terminal epitope does not significantly interfere with secretion because T4S signals are often located in the C-terminal protein region (Vergunst et al., 2000; Nagai et al., 2005). When analyzed in strain 85-10, epitope-tagged derivatives of XCV3751, XCV1120 and XCV0332 were stably synthesized. In contrast, c-Myc-TraI was not detected in cell extracts by immunoblotting, suggesting that it was unstable when expressed under control of the *lac* promoter (data not shown). *traI* was, therefore, expressed under control of a native promoter from *X. euvesicatoria*, which resulted in detectable protein synthesis (see below). For *in vitro* secretion assays, bacteria were cultivated in NYG medium containing BSA which likely stabilizes secreted proteins as was shown for substrates of the T3S system (Rossier et al., 1999). Total cell extracts and culture supernatants were separated by filtration and analyzed by immunoblotting. Notably, all tested proteins were detected in the culture supernatant of strain 85-10, suggesting that they were secreted (**Figures 9A, B**).

To investigate whether secretion of the analyzed proteins was dependent on the T4S systems, we performed *in vitro* secretion assays with strain 85-10Δ*icmEΔvirB4*, which lacks predicted essential components of the VirB/VirD4 and Icm/Dot T4S system. Secretion of c-Myc-XCV1120 and c-Myc-XCV3751 was unaffected in strain 85-10Δ*icmEΔvirB4*, suggesting that the presence of the XVIPCD and the interaction with VirD4 is not sufficient to exclusively target both proteins to the T4S systems (**Figure 9A**). In contrast, significantly reduced amounts of c-Myc-XCV0332 and c-Myc-TraI were detected in the culture supernatant of strain 85-10Δ*icmEΔvirB4*, indicating a contribution of the T4S systems to secretion (**Figure 9B**). As a control, all supernatants were incubated in the presence of proteinase K to investigate a possible contribution of OM vesicles to secretion. OM vesicles were previously shown to mediate secretion of degradative enzymes from *X. euvesicatoria* (Solé et al., 2015). Detectable secretion of all tested candidate T4S substrates, however, was abolished upon treatment of the culture supernatants with proteinase K, suggesting that the secreted proteins were not present in OM vesicles (**Figures 9A, B**).

To further elucidate the contribution of the VirB/VirD4 or the Icm/Dot T4S system to secretion of c-Myc-XCV0332 and c-Myc-TraI, we introduced the corresponding expression constructs into *virB4* and *icmE* single deletion mutants. Secretion of c-Myc-TraI was unaffected in both strains, suggesting that it is secreted by either the VirB/VirD4 or the Icm/Dot T4S system (**Figure 9C**). In contrast, secretion of c-Myc-XCV0332 was reduced but not completely abolished in strains 85-10Δ*icmE* and 85-10Δ*virB4* (**Figure 9D**). We, therefore, assume that the VirB/VirD4 and Icm/Dot T4S systems both contribute to the efficient secretion of XCV0332, which was abolished in the absence of both functional T4S systems (**Figure 9B**). Wild-type secretion levels of c-Myc-XCV0332 were restored when *virB4* and *icmE* were reinserted into the genomes of strains 85-10Δ*virB4* and 85-10Δ*icmE*, respectively (**Figure 9D**). This suggests that reduced secretion of XCV0332 in the single mutants was specifically caused by the absence of VirB4 and IcmE, respectively, and not by a polar effect of the deletions on other genes. Our findings thus indicate that VirB4/VirD and Icm/Dot T4S systems are involved in *in vitro* protein secretion and that XCV0332 and TraI are targeted to both T4S systems. In case of TraI, the Icm/Dot T4S system appears to fully compensate for the loss of a functional VirB/VirD4 T4S system.

## Discussion

*X. euvesciatoria* is the only known plant-pathogenic bacterium which contains a predicted Icm/Dot T4S system in addition to the VirB/VirD4 T4S system. The corresponding T4S gene clusters are located on plasmids pXCV138 and pXCV38, respectively, in *X. euvesicatoria* strain 85-10 (Thieme et al., 2005). Additional *virB* genes as well as *virD4* are present on the chromosome, however, a complete chromosomal *virB/virD4* T4S gene cluster as described for *X. axonopodis* pv. *citri* is missing in *X. euvesicatoria*. Given that the role of T4S systems from *X. euvesicatoria* has not yet been investigated, we performed functional studies with T4S genes and analyzed secretion of candidate T4S substrates and their interaction with the T4S systems. The analysis of promoter-reporter fusions suggests that *vir* and *icm/dot* genes are expressed *in vitro* and *in planta*. In agreement with this finding, several Vir proteins including VirD4 and VirB proteins encoded on plasmid pXCV38 were previously identified in *X. euvesicatoria* cell extracts by mass spectrometry (Abendroth et al., 2017). Interestingly, the chromosomal *virD4* gene, which encodes the predicted T4CP of the VirB/VirD4 T4S system, appears to be predominantly expressed *in vitro*. Given that VirD4 is an essential component of T4S systems, the reduced *in planta* promoter activity of *virD4* indicates that T4S does not significantly contribute to the interaction of *X. euvesicatoria* with its host plants. This hypothesis is supported by the finding that deletion of predicted essential genes of both T4S systems did not interfere with disease symptom formation and *in planta* growth of *X. euvesicatoria.* In agreement with our observations, a virulence function or contribution to the host-pathogen interaction has not been attributed to any T4S system from *Xanthomonas* spp. (El Yacoubi et al., 2007; He et al., 2007; Souza et al., 2011; Jacob et al., 2014).

The chromosomally encoded VirB/VirD4 T4S system from *X. axonopodis* pv. *citri* was previously shown to deliver toxins into bacterial target cells and thus acts as a protein translocation machine (Souza et al., 2015). A similar function was reported for the VirB/VirD4 T4S system from the related opportunistic human pathogen *S. maltophilia*, suggesting a function of T4S systems as bacterial killing devices (Bayer-Santos et al., 2019). Components of the chromosomally encoded VirB/VirD4 T4S system from *X. axonopodis* pv. *citri* are homologous to corresponding proteins encoded on the chromosome of *X. euvesicatoria.* However, the chromosomal gene cluster in *X. euvesicatoria* is incomplete and might thus have resulted from chromosomal rearrangements, leading to the loss of function of the corresponding VirB/VirD4 T4S system. In agreement with this hypothesis, we did not detect a toxic effect of *X. euvesicatoria* T4S systems on *E. coli* strains.

Notably, *X. axonopodis* pv. *citri* and *X. euvesicatoria* contain a second *virB* gene cluster on plasmids pXAC64 and pXCV38, respectively. The association of the plasmid-localized T4S gene clusters with *traI* and *traG* genes suggests a role in conjugation which remains to be demonstrated in *X. axonopodis* pv. *citri*. The predicted functional differences of plasmid- and chromosomally encoded T4S systems as possible conjugation machines and toxin translocators, respectively, are reflected by the low sequence similarities of the corresponding VirB proteins. Interestingly, VirD4 is encoded as a single copy gene on the chromosome and might thus contribute to secretion by both VirB/VirD4 T4S systems in *X. axonopodis* pv. *citri*, suggesting that it interacts with a broad spectrum of substrates.

Plasmid transfer in *Xanthomonas* spp. was previously shown to occur *in vitro* and *in planta*, and leads to the exchange of virulence factors, thus potentially generating strains with altered host range or pathogenic potential (Basim et al., 1999; El Yacoubi et al., 2007; Wang et al., 2007). Mobilizable plasmids often carry the genes required for horizontal gene transfer such as plasmid pXcB from *X. citri* pv. *citri*, which encodes components of a VirB/VirD4 T4S system (El Yacoubi et al., 2007; Virolle et al., 2020). Mutation of *virB4* affects self-transmission of pXcB from *E. coli*, thus confirming a role of the VirB/VirD4 T4S system in conjugation (El Yacoubi et al., 2007). When analyzed in *X. citri*, however, self-transmission of pXcB is unaffected in the absence of VirB4, suggesting the contribution of redundant plasmid transfer functions outside of pXcB which compensate for the loss of VirB4 (El Yacoubi et al., 2007). Similarly to pXcB, plasmid pXCV38 is transferable between different *X. euvesicatoria* strains as was demonstrated using a derivative of pXCV38 from strain 82-8 with a spectinomycin resistance gene. Deletion of the chromosomal *virD4* gene in the donor strain significantly reduced plasmid transfer, indicating that the VirB/VirD4 T4S system from *X. euvesicatoria* acts as a conjugation machine. In strains lacking VirB4, however, residual plasmid transfer was observed, suggesting the contribution of functionally redundant proteins which partially compensate the loss of VirB4 as was described for *X. citri* pv. *citri virB4* mutants (El Yacoubi et al., 2007). It is possible that a *virB4* homolog on plasmid XCV38_82-8_ partially promotes plasmid transfer in *virB4* mutants. In future studies, genome and plasmid sequence analyses are required to investigate the presence of T4S gene clusters in strain 82-8.

In agreement with their role in conjugation, VirB proteins assemble into heterooligomeric complexes corresponding to known subassemblies of T4S systems as was suggested by the results of protein-protein interaction studies. BACTH assays detected homo- and heterooligomerization of the predicted core components VirB7 and VirB10, which is in agreement with known assemblies of homologous proteins in T4S systems. As was shown for the conjugative T4S system encoded on plasmid pKM101 and the VirB/VirD4 T4S system from *X. axonopodis* pv. *citri*, a complex of 14 copies of VirB10 spans both bacterial membranes and associates in the OM layer with 14 copies of VirB9 and VirB7 (Sgro et al., 2018; Sheedlo et al., 2022). Interactions between VirB7 and VirB9 and the self-interaction of VirB7 are required for T4S system assembly and activity (Oliveira et al., 2016). VirB7 proteins are usually small lipoproteins of approximately 40 amino acids. In *Xanthomonas* spp., however, VirB7 proteins contain between 130 and 185 amino acids due to the presence of an additional globular C-terminal domain, designated N0 domain, which likely mediates VirB7 oligomerization and thus promotes the assembly of the T4S system (Sgro et al., 2019). Cryo-electron microscopy studies of a VirB7/VirB9/VirB10 complex from *X. axonopodis* pv. *citri* revealed that the N0 domain assembles as extra peripheral ring of the VirB7-VirB9-VirB10 OM complex (Souza et al., 2011; Sgro et al., 2019). Interestingly, however, in *X. euvesicatoria* and *X. axonopodis* pv. *citri*, chromosomally and plasmid-encoded VirB7 proteins share limited amino acid sequence similarities, which possibly reflects structural differences of translocation- and conjugation-associated X-T4S systems.

In addition to the interactions between VirB7 and VirB10, BACTH assays revealed interactions between VirB10 and the three predicted ATPases VirD4, VirB4 and VirB11 which homo- and/or heterooligomerize as expected. Furthermore, IcmE, which corresponds to VirB10, interacts with all three predicted ATPases DotL, DotO and DotB of the predicted Icm/Dot T4S system. We, therefore, assume that Vir and Icm/Dot proteins assemble into heterooligomeric complexes, most likely corresponding to T4S systems. Notably, however, the *icm/dot* T4S gene cluster on plasmid pXCV183 only contains 16 genes which encode the predicted essential core components of the secretion apparatus whereas ten genes present in the *icm/dot* gene cluster from *L. pneumophila* are absent in *X. euvesicatoria*. The contribution of *icm/dot* genes to *in vitro* T4S in *X. euvesicatoria*, however, suggests that the *icm/dot* T4S gene cluster encodes a functional T4S system for protein delivery. Given that *icm/dot* genes do not significantly contribute to virulence of *X. euvesicatoria* and plasmid transfer (data not shown), a possible contribution to protein delivery into bacterial or eukaryotic cells remains to be investigated.

Substrates of T4S systems, so-called X-Tfes, were previously identified from *X. axonopodis* pv. *citri* based on their interactions with the T4CP VirD4 which was used as a bait in yeast two-hybrid screens (Alegria et al., 2005). The identified XVIPs contain a C-terminal domain, designated XVIPCD, of approximately 120 amino acids which provides the binding site for VirD4 (Oka et al., 2022). Additional XVIPCD-containing proteins were identified by bioinformatic analyses and often share similarities with peptidoglycan-modifying enzymes, suggesting a function in the bacterial periplasm (Sgro et al., 2019). X-Tfes are likely delivered into bacterial target cells where they presumably cause lysis as was shown for the *X. axonopodis* pv. *citri* X-Tfe XAC2609 (Souza et al., 2015). In the present study, we analyzed homologous proteins from *X. euvesicatoria* with C-terminal XVIPCDs as well as the predicted relaxase TraI, which is a known substrate of conjugation-associated VirB/VirD4 T4S systems and lacks an XVIPCD. All candidates including TraI interacted with VirD4, suggesting that substrate binding to VirD4 is not exclusively linked to the presence of an XVIPCD.

The N-terminal region of the XVIPCD adopts an ααβββ fold which interacts with the C-terminal AAD from VirD4 as was shown by previous solution NMR spectroscopy studies of the X-Tfe XAC2609 (Oka et al., 2022). In contrast, the C-terminal glutamine-rich tail of the XVIPCD is dispensable for the interaction with the AAD from VirD4 (Oka et al., 2022). Interestingly, structure predictions revealed similar ααβββ motifs in the C-terminal regions of the XVIPCD-containing candidate substrates XCV0332, XCV1120 and XCV3571 from *X. euvesicatoria*. Yet, a contribution of the XVIPCD of candidate X-Tfes from *X. euvesicatoria* to the interaction with VirD4 remains to be investigated. Notably, the analyzed candidate substrates XCV3571 and XCV1120 were secreted by wild-type and T4S mutant strains, suggesting that the presence of the XVIPCD and the interaction with VirD4 is not sufficient for the exclusive targeting of proteins to the T4S system. This is in agreement with the finding that the ααβββ motif in XAC2609 from *X. axonopodis* pv. *citri* is required for initial substrate recognition but is presumably not sufficient for T4S-dependent protein delivery (Oka et al., 2022). It was proposed that the C-terminal protein region outside the conserved XVIPCD promotes translocation, possibly by interacting with a yet unidentified adaptor protein of the T4S system (Oka et al., 2022). The presence of an additional translocation signal outside the XVIPCD in T4S substrates from *X. euvesicatoria* is supported by the finding that TraI is secreted by the T4S systems despite the lack of an XVIPCD.

Taken together, our *in vitro* secretion assays led to the identification of TraI and XCV0332 as T4S substrates from *X. euvesicatoria*. XCV0332 is a homolog of the X-Tfe XAC0323 and mainly consist of the XVIPCD. Both proteins might thus represent parts of ancient X-Tfes which result from mutations leading to the loss of the N-terminal protein region (Sgro et al., 2019). Secretion of XCV0332 was abolished in the absence of functional VirB/VirD4 and Icm/Dot T4S systems whereas *icmE* and *virB4* single mutants led to reduced but still detectable secretion of XCV0332. We, therefore, assume that both T4S systems additively contribute to XCV0332 secretion, indicating that XVIPCD-containing proteins can be targeted to both T4S systems in *X. euvesicatoria*. A similar finding was observed for the secretion of TraI. In contrast to XCV0332, however, secretion of TraI was unaffected in *icmE* and *virB4* single mutants. This suggests that the presence of either the VirB/VirD4 or the Icm/Dot T4S system is sufficient to promote TraI secretion at wild-type levels. The dual targeting of TraI was unexpected and points to a broad substrate specificity of both T4S systems. In future studies, we will, therefore, investigate possible interactions of TraI with components of the Icm/Dot T4S system as well as the biological significance of Icm/Dot-mediated protein delivery. Notably, in preliminary experiments, we did not detect any contribution of the Icm/Dot T4S system to plasmid transfer. However, given that mobilizable plasmids often contain T4S genes which are involved in plasmid transfer, a possible contribution of the Icm/Dot T4S system to the transfer of plasmid pXCV183, which contains the *icm/dot* T4S genes, needs to be analyzed in future experiments.

In summary, our study showed that *X. euvesicatoria* contains two functional T4S systems which contribute to protein secretion and presumably do not promote toxin delivery into *E. coli*. The VirB/VirD4 T4S system acts as a conjugation system for plasmid transfer between *X. euvesicatoria* strains and shares substrate specificity with the Icm/Dot system which serves as additional protein delivery system. The contribution of the Icm/Dot T4S system to the secretion of additional T4S effectors and a possible protein transport into plant cells remains to be investigated.

## Data availability statement

The original contributions presented in the study are included in the article/supplementary material. Further inquiries can be directed to the corresponding author.

## Author contributions

SD, FS and DB conceived and designed the experiments. SD and FS performed the experiments and analyzed the data. SD and DB wrote the manuscript. All authors contributed to the article and approved the submitted version.
