# Supplementary material for: Functional characterization of VirB/VirD4 and Icm/Dot type IV secretion systems from the plant-pathogenic bacterium Xanthomonas euvesicatoria
Source: Front Cell Infect Microbiol. 2023 Aug 1;13:1203159. doi: 10.3389/fcimb.2023.1203159 (PMC10432156; doi:10.3389/fcimb.2023.1203159)
Supplement: Supplementary file 1 [file DataSheet_1.pdf]

Supplementary Material

Functional characterization of VirB/VirD4 and Icm/Dot type IV secretion systems from the plant-pathogenic bacterium *Xanthomonas euvesicatoria*

Sabine Drehkopf[1], Felix Scheibner[2] and Daniela Büttner[1,*]

* Correspondence:
daniela.buettner@genetik.uni-halle.de

Supplemental tables:

Table S1: Bacterial strains and plasmids used in this study.

Table S2: Primers used in this study.

Table S3: Accession numbers and predicted functions of VirB/VirD4 proteins from *A. tumefaciens* and *Xanthomonas* spp.

Table S4: Sequences of predicted *vir* and *icm/dot* promoters from *X. euvesicatoria*.

Table S5: Accession numbers and predicted functions of Icm/Dot proteins from *L. pneumophila.*

Table S6: Transfer of plasmid pXCV38$_{82\text{-}8}$::*spec*^{R} from strain 82-8 into the recipient strain 85-10::*gent*^{R}.

Table S7: XVIPCDs in T4S candidate substrates from *X. euvesicatoria.*

Supplemental figure:

Figure S1: Synthesis and immunological detection of T18 and T25 fusion proteins in *E. coli* strain JM109.

Figure S2: Results of BACTH assays with TraG from *X. euvesicatoria.*

.

Table S1: Bacterial strains and plasmids used in this study

Strain or plasmid	Relevant characteristics[1]	Reference or source
Xanthomonas euvesicatoria		
85-10	Pepper-race 2; wild type; Rif^r	Canteros, 1990; Kousik and Ritchie, 1998
85-10Δ*virB4*	*virB4* deletion mutant of strain 85-10	This study
85-10Δ*virD4*	*virD4* deletion mutant of strain 85-10	This study
85-10Δ*virG*	*virG* deletion mutant of strain 85-10	This study
85-10Δ*virA*	*virA* deletion mutant of strain 85-10	This study
85-10Δ*virB11*Δ*virB1*	*virB11* and *virB1* deletion mutant of strain 85-10	This study
85-10Δ*virD4*Δ*virB11*Δ*virB1*	*virD4*, *virB11* and *virB1* deletion mutant of strain 85-10	This study
85-10 Δ*virD4*Δ*virB11*Δ*virB1*Δ*virB4*	Derivative of strain 85-10 deleted in *virD4*, *virB11*, *virB1* and *virB4*	This study
85-10 Δ*virB6*Δ*virB8*Δ*virB9*Δ*virB10*	Derivative of strain 85-10 deleted in *virB6*, *virB8*, *virB9* and *virB10*	This study
85-10 Δ*virB6*Δ*virB8*Δ*virB9*Δ*virB10* *ΔicmEΔdotCB*	Derivative of strain 85-10 deleted in *virB6*, *virB8*, *virB9*, *virB10*, *icmE*, *dotC* and *dotB*	This study
85-10Δ*dotCB*	Derivative of strain 85-10 deleted in *dotC* and *dotB*	This study
85-10Δ*icmE*	*icmE* deletion mutant of strain 85-10	This study
85-10Δ*dotCB*Δ*icmE*	Derivative of strain 85-10 deleted in *dotC*, *dotB* and *icmE*	This study
85-10Δ*virB4*Δ*icmE*	Derivative of strain 85-10 deleted in *virB4* and *icmE*	This study
85-10Δ*dotCB*Δ*icmE*Δ*virB4*	Derivative of strain 85-10 deleted in *dotC*, *dotB*, *icmE* and *virB4*	This study
85-10::*kan*^{R}	Insertion mutant of 85-10 carrying *gfp* and the kanamycin resistance gene under control of the gentamycin resistance promoter of pBRM on pXCV183; Rif^r, Km^r	This study
85-10Δ*xopQ*	*xopQ* deletion mutant of strain 85-10	Adlung et al., 2016
82-8	Pepper-race 1; wild type; Rif^r	Bonas et al., 1989
85-10::*gent*^{R}	Insertion mutant of 85-10 carrying the gentamycin resistance gene under control of the *lac* promoter in the *hpaFG* region; Rif^r, Gm^r	This study
85-10Δ*virD4*::*gent*^{R}	Insertion mutant of 85-10Δ*virD4* carrying the gentamycin resistance gene under control of the *lac* promoter in the *hpaFG* region; Rif^r, Gm^r	This study
85-10Δ*virB4*::*gent*^{R}	Insertion mutant of 85-10Δ*virB4* carrying the gentamycin resistance gene under control of the *lac* promoter in the *hpaFG* region; Rif^r, Gm^r	This study
E. coli		
OneShot®TOP10	F$^-$ *mcrA* Δ(*mrr-hsdRMS-mcrBC*) φ80*lac*ZΔM15 Δ*lac*X74 *rec*A1 *ara*Δ139 Δ(*ara-leu*)7697 *galU galK rpsL endA1 nupG* (Str^r)	Invitrogen
Dh5α λpir	F$^-$ *recA hsdR17*(r_k^-,m_k^+) *ϕ80dlacZ DM15 [λpir]*	Ménard et al., 1993
BL21(DE3)	F$^-$ *ompt hsdS20*($r_b^-m_b^-$) *gal*	Stratagene
Rosetta DE3 pLysS		
JM109	F$^-$, *traD36 proA+B+ lacIq Δ(lacZ)M15/ Δ(lac-proAB) glnV44 e14- gyrA96 recA1 relA1 endA1 thi hsdR17*	Yanisch-Perron et al., 1985
BTH101	F$^-$, *cya-99, araD139, galE15, galK16, rpsL1 (StrR), hsdR2, mcrA1, mcrB1*	Euromedex; Battesti and Bouveret, 2012
**Plasmids**		
pRK2013	ColE1 replicon, TraRK$^+$ Mob$^+$; Km^r	Figurski and Helinski, 1979
pBBR1MCS-5	Broad-host-range vector; *lac* promoter; Gm^r	Kovach et al., 1995
pBRM	Golden Gate-compatible derivative of pBBR1MCS-5	Szczesny et al., 2010
pBRM-P	Golden Gate-compatible derivative of pBBR1MCS-5 without promoter	Szczesny et al., 2010

pBRM-P(stop)	Golden Gate-compatible derivative of pBRM-P with a stop codon in the 3' region	Hausner et al., 2013
pBRNM	Golden Gate-compatible derivative of pBBR1MCS-5 with 3 x c-Myc-encoding sequence in the 5' region of the inserted gene and a stop codon in the 3' region; Gm^r	This study
pBRM-P+T	Golden Gate-compatible derivative of pBBR1MCS-5 without promoter upstream of the cloning sites and an *rrnb T1* terminator	Helm and Schmidtke, unpublished
pDSK602	Broad-host-range vector containing a *lacUV5* promoter; Sm^r	Murillo et al., 1994
pICH41021	Derivative of pUC19 with mutated *BsaI* site; Ap^r	Hausner et al., 2019
pUC57	Classical cloning vector with mutated *BsaI* site; Ap^r	Thermo Fisher Scientific
pLAND	Derivative of pOK1 containing fragments of the *hpaFG* region, the *lac* promoter and a triple *c-Myc* epitope-encoding sequence; Sm^r	Lorenz et al., 2012
pLAND-P	Derivative of pOK1 containing fragments of the *hpaFG* region, a triple *c-Myc* epitope-encoding sequence and lacking a promoter upstream of the *Bsa*I sites; Sm^r	Lorenz et al., 2012
pOGG2	Golden Gate-compatible derivative of suicide vector pOK1, *sacB sacQ mobRK2 oriR6K*; Sm^r	Schulze et al., 2012
pAGM9121	pUC19-derived vector, *lacZα* fragment flanked by *BpiI* sites (CTCA/CGAG); GGAG/CGCT *BsaI* fusion sites; Sm^r	Addgene #51833; Weber et al., 2011
pICH47732	Derived from pUC19, level 1 for promoters; *lacZα* fragment flanked by *BsaI* sites (GGAG/CGCT); TGCC/GCAA *BpiI* fusion sites; Ap^r	Addgene #48000; Weber et al., 2011
pICH79631	Derived from pICH50251, containing dTALE-2 with stop-codon for position 2 in level M; GCAA/ACTA *BpiI* fusion sites; Ap^r	Gift from S. Marillonnet
pICH50122	Derived from pUC19, end linker for position 3 contains a terminator sequence; ACTA/ GGGA *BpiI* fusion sites; Ap^r	Gift from S. Marillonnet
pICH77739	Level 2 vector derived from pBIN19, RK2 ori, *lacZα* fragment flanked by *BpiI* sites; Km^r	Weber et al., 2011
$pKT25_{GG}$	Golden Gate-compatible derivative of pKT25 encoding the T25 fragment downstream of a *lac* promoter and in frame with a C-terminal FLAG epitope-encoding sequence; contains *lacP-eforRed* flanked by *Bsa*I sites downstream of the *T25-FLAG* fragment; Km^r	Otten and Büttner, 2021
$pKNT25_{GG}$	Golden Gate-compatible derivative of pKNT25, encodes the T25 fragment in frame with an N-terminal FLAG epitope-encoding sequence downstream of the *lac* promoter, contains *lacP-eforRed* flanked by *Bsa*I sites upstream of the *T25-FLAG* fragment; Km^r	Otten and Büttner, 2021
$pUT18_{GG}$	Golden Gate-compatible derivative of pUT18 containing *lacP-eforRed* flanked by *Bsa*I sites upstream of the *FLAG-T18* fragment; Gm^R	Otten and Büttner, 2021
$pUT18C_{GG}$	Golden Gate-compatible derivative of pUT18C containing *lacP-eforRed* flanked by *Bsa*I sites downstream of the *T18-FLAG* fragment; Gm^R	Otten and Büttner, 2021
pICH41021-$virD4_{1-950}$	Derivative of pICH41021 containing bp 1 - 950 of *virD4* flanked by *BsaI* sites with TATG and GCCT overhangs	This study
pICH41021-$virD4_{951-1450}$	Derivative of pICH41021 containing bp 951 - 1450 of *virD4* flanked by *BsaI* sites with GCCT and GTCA overhangs	This study
pICH41021-$virD4_{1451-1671}$	Derivative of pICH41021 containing bp 1451 – 1671 of *virD4* flanked by *BsaI* sites with GTCA and GGTG overhangs	This study

pUC57-XCV4361$_{prom}$	Derivative of pUC57 containing the putative promoter of XCV4361 flanked by *BsaI* sites with ATTC and TATG overhangs	This study
pICH41021-traI	Derivative of pICH41021 containing *traI* flanked by *BsaI* sites with TATG and GGTG fusion sites	This study
pUC57-XCV0332	Derivative of pUC57 containing XCV0332 flanked by *BsaI* sites with TATG and GGTG fusion sites	This study
pICH41021-XCV3751	Derivative of pICH41021 containing *XCV3751* flanked by *BsaI* sites with TATG and GGTG fusion sites	This study
pUC57-XCV1120	Derivative of pUC57 containing *XCV1120* flanked by *BsaI* sites with TATG and GGTG fusion sites	This study
pICH41021-virB10	Derivative of pICH41021 containing *virB10* flanked by *BsaI* sites with TATG and GGTG fusion sites	This study
pICH41021-virB11	Derivative of pICH41021 containing *virB11* flanked by *BsaI* sites with TATG and GGTG fusion sites	This study
pICH41021- virB7	Derivative of pICH41021 containing *virB7* flanked by *BsaI* sites with TATG and GGTG fusion sites	This study
pICH41021-dotB	Derivative of pICH41021 containing *dotB* flanked by *BsaI* sites with TATG and GGTG fusion sites	This study
pICH41021-dotO$_{1-109}$	Derivative of pICH41021 containing bp 1 - 109 of *dotO* flanked by *BsaI* sites with TATG and AGAC overhangs	This study
pICH41021-dotO$_{110-3048}$	Derivative of pICH41021 containing bp 110 - 3048 of *dotO* flanked by *BsaI* sites with AGAC and GGTG overhangs	This study
pICH41021-icmE$_{1-645}$	Derivative of pICH41021 containing bp 1 - 645 of *icmE* flanked by *BsaI* sites with TATG and ACGA overhangs	This study
pICH41021-icmE$_{646-796}$	Derivative of pICH41021 containing bp 646 - 796 of *icmE* flanked by *BsaI* sites with ACGA and GACG overhangs	This study
pICH41021-icmE$_{797-1248}$	Derivative of pICH41021 containing bp 797 - 1248 of *icmE* flanked by *BsaI* sites with GACG and GGTG overhangs	This study
pICH41021-dotL$_{1-1460}$	Derivative of pICH41021 containing bp 1 - 1460 of *dotL* flanked by *BsaI* sites with TATG and ACCG overhangs	This study
pICH41021-dotL$_{1461-2001}$	Derivative of pICH41021 containing bp 1461 - 2001 of *dotL* flanked by *BsaI* sites with ACCG and GCGG overhangs	This study
pICH41021-dotL$_{2002-2568}$	Derivative of pICH41021 containing bp 2002 - 2568 of *dotL* flanked by *BsaI* sites with GCGG and GGTG overhangs	This study
pXCV38$_{82-8}$::*spec*^{R}	Derivative of the native plasmid pXCV38 from *X. euvesicatoria* strain 82-8 containing a spectinomycin resistance gene inserted in *avrBs3*	Bonas et al., 1989
**Expression constructs and suicide vectors**		
pDSF300	Derivative of pDSK602 encoding AvrBs3	Van den Ackerveken et al., 1996
pBRNM-XCV3751	Derivative of pBRNM encoding c-Myc-XCV3751 under control of the *lac* promoter	This study
pBRNM-XCV1120	Derivative of pBRNM encoding c-Myc-XCV1120 under control of the *lac* promoter	This study
pBRNM-XCV0332	Derivative of pBRNM encoding c-Myc-XCV0332 under control of the *lac* promoter	This study
pBRM-P(stop)-XCV4361$_{prom}$-c-Myc-traI	Derivative of pBRM-P(stop) encoding c-Myc-TraI under control of the putative *XCV4361* promoter	This study
pLAND-virB4	Derivative of pLAND containing *virB4*	This study
pLAND-P-XCV0160$_{prom}$-icmE	Derivative of pLAND-P containing *icmE* under the control of the XCV0160 promoter	This study
pLAND-gent^R	Derivative of pLAND containing the gentamycin resistance gene cassette from vector pBRM	This study

pOGG2-kan^{R}gfp(pXCV183)	Derivative of pOGG2 carrying the flanking sequences of nucleotide position 2831 in pXCV183 and containing the kanamycin resistance and the *gfp* gene	This study
pOGG2-ΔvirB4	Derivative of pOGG2 carrying flanking sequences of *virB4*	This study
pOGG2-ΔvirD4	Derivative of pOGG2 carrying flanking sequences of *virD4*	This study
pOGG2-ΔvirG	Derivative of pOGG2 carrying flanking sequences of *virG*	This study
pOGG2-ΔvirA	Derivative of pOGG2 carrying flanking sequences of *virA*	This study
pOGG2-ΔvirB11ΔvirB1	Derivative of pOGG2 carrying flanking sequences of *virB11* and *virB1*	This study
pOGG2-ΔvirB6ΔvirB8ΔvirB9ΔvirB10	Derivative of pOGG2 carrying flanking sequences of *virB6* and *virB10*	This study
pOGG2-ΔicmE	Derivative of pOGG2 carrying flanking sequences of *icmE*	This study
pOGG2-ΔdotCB	Derivative of pOGG2 carrying flanking sequences of *dotC* and *dotB*	This study
**BACTH constructs**		
pUT18$_{GG}$-virD4	Derivative of pUT18$_{GG}$ encoding VirD4-FLAG-T18	This study
pUT18C$_{GG}$-virD4	Derivative of pUT18C$_{GG}$ encoding T18-FLAG-VirD4	This study
pKNT25$_{GG}$-virD4	Derivative of pKNT25-GG encoding VirD4-FLAG-T25	This study
pKT25$_{GG}$-virD4	Derivative of pKT25$_{GG}$ encoding T25-FLAG-VirD4	This study
pUT18$_{GG}$-dotL	Derivative of pUT18$_{GG}$ encoding DotL-FLAG-T18	This study
pUT18C$_{GG}$-dotL	Derivative of pUT18C$_{GG}$ encoding T18-FLAG-DotL	This study
pKNT25$_{GG}$-dotL	Derivative of pKNT25-GG encoding DotL-FLAG-T25	This study
pKT25$_{GG}$-dotL	Derivative of pKT25$_{GG}$ encoding T25-FLAG-DotL	This study
pUT18$_{GG}$-XCV3751	Derivative of pUT18$_{GG}$ encoding XCV3751-FLAG-T18	This study
pUT18C$_{GG}$-XCV3751	Derivative of pUT18C$_{GG}$ encoding T18-FLAG-XCV3751	This study
pKNT25$_{GG}$-XCV3751	Derivative of pKNT25-GG encoding XCV3751-FLAG-T25	This study
pKT25$_{GG}$-XCV3751	Derivative of pKT25$_{GG}$ encoding T25-FLAG-XCV3751	This study
pUT18$_{GG}$-XCV0332	Derivative of pUT18$_{GG}$ encoding XCV0332-FLAG-T18	This study
pUT18C$_{GG}$-XCV0332	Derivative of pUT18C$_{GG}$ encoding T18-FLAG-XCV0332	This study
pKNT25$_{GG}$-XCV0332	Derivative of pKNT25-GG encoding XCV0332-FLAG-T25	This study
pKT25$_{GG}$-XCV0332	Derivative of pKT25$_{GG}$ encoding T25-FLAG-XCV0332	This study
pUT18$_{GG}$-XCV1120	Derivative of pUT18$_{GG}$ encoding XCV1120-FLAG-T18	This study
pUT18C$_{GG}$-XCV1120	Derivative of pUT18C$_{GG}$ encoding T18-FLAG-XCV1120	This study
pKNT25$_{GG}$-XCV1120	Derivative of pKNT25-GG encoding XCV1120-FLAG-T25	This study
pKT25$_{GG}$-XCV1120	Derivative of pKT25$_{GG}$ encoding T25-FLAG-XCV1120	This study
pUT18$_{GG}$-traI	Derivative of pUT18$_{GG}$ encoding TraI-FLAG-T18	This study
pUT18C$_{GG}$-traI	Derivative of pUT18C$_{GG}$ encoding T18-FLAG-TraI	This study
pKNT25$_{GG}$-traI	Derivative of pKNT25-GG encoding TraI-FLAG-T25	This study
pKT25$_{GG}$-traI	Derivative of pKT25$_{GG}$ encoding T25-FLAG-TraI	This study
pUT18$_{GG}$-virB10	Derivative of pUT18$_{GG}$ encoding VirB10-FLAG-T18	This study
pUT18C$_{GG}$-virB10	Derivative of pUT18C$_{GG}$ encoding T18-FLAG-VirB10	This study
pKNT25$_{GG}$-virB10	Derivative of pKNT25-GG encoding VirB10-FLAG-T25	This study
pKT25$_{GG}$-virB10	Derivative of pKT25$_{GG}$ encoding T25-FLAG-VirB10	This study
pUT18$_{GG}$-virB11	Derivative of pUT18$_{GG}$ encoding VirB11-FLAG-T18	This study
pUT18C$_{GG}$-virB11	Derivative of pUT18C$_{GG}$ encoding T18-FLAG-VirB11	This study
pKNT25$_{GG}$-virB11	Derivative of pKNT25-GG encoding VirB11-FLAG-T25	This study
pKT25$_{GG}$-virB11	Derivative of pKT25$_{GG}$ encoding T25-FLAG-VirB11	This study
pUT18$_{GG}$-virB7	Derivative of pUT18$_{GG}$ encoding VirB7-FLAG-T18	This study

pUT18C$_{GG}$-virB7	Derivative of pUT18C$_{GG}$ encoding T18-FLAG-VirB7	This study
pKNT25$_{GG}$-virB7	Derivative of pKNT25-GG encoding VirB7-FLAG-T25	This study
pKT25$_{GG}$-virB7	Derivative of pKT25$_{GG}$ encoding T25-FLAG-VirB7	This study
pUT18$_{GG}$-dotB	Derivative of pUT18$_{GG}$ encoding DotB-FLAG-T18	This study
pUT18C$_{GG}$-dotB	Derivative of pUT18C$_{GG}$ encoding T18-FLAG-DotB	This study
pKNT25$_{GG}$-dotB	Derivative of pKNT25-GG encoding DotB-FLAG-T25	This study
pKT25$_{GG}$-dotB	Derivative of pKT25$_{GG}$ encoding T25-FLAG-DotB	This study
pUT18$_{GG}$-dotO	Derivative of pUT18$_{GG}$ encoding DotO-FLAG-T18	This study
pKNT25$_{GG}$-dotO	Derivative of pKNT25-GG encoding DotO-FLAG-T25	This study
pKT25$_{GG}$-dotO	Derivative of pKT25$_{GG}$ encoding T25-FLAG-DotO	This study
pUT18$_{GG}$-icmE	Derivative of pUT18$_{GG}$ encoding IcmE-FLAG-T18	This study
pUT18C$_{GG}$-icmE	Derivative of pUT18C$_{GG}$ encoding T18-FLAG-IcmE	This study
pKNT25$_{GG}$-icmE	Derivative of pKNT25-GG encoding IcmE-FLAG-T25	This study
pKT25 $_{GG}$-icmE	Derivative of pKT25$_{GG}$ encoding T25-FLAG-IcmE	This study
**Constructs for promoter-reporter assays**		
pICH41021-virB2$_{prom}$	Derivative of pICH41021 containing the putative promoter of *virB2* flanked by *BsaI* sites with ATTC and TATG overhangs for insertion into pBRM-P	This study
pICH41021-virB5$_{prom}$	Derivative of pICH41021 containing the putative promoter of *virB5* flanked by *BsaI* sites with ATTC and TATG overhangs for insertion into pBRM-P	This study
pICH41021-virD4$_{prom}$	Derivative of pICH41021 containing the putative promoter of *virD4* flanked by *BsaI* sites with ATTC and TATG overhangs for insertion into pBRM-P	This study
pICH41021-virG$_{prom}$	Derivative of pICH41021 containing the putative promoter of *virG* flanked by *BsaI* sites with ATTC and TATG overhangs for insertion into pBRM-P	This study
pICH41021-dotA$_{prom}$	Derivative of pICH41021 containing the putative promoter of *dotA* flanked by *BsaI* sites with ATTC and TATG overhangs for insertion into pBRM-P	This study
pICH41021-dotD$_{prom}$	Derivative of pICH41021 containing the putative promoter of *dotD* flanked by *BsaI* sites with ATTC and TATG overhangs for insertion into pBRM-P	This study
pICH41021-icmL$_{prom(229)}$	Derivative of pICH41021 containing 229 bp upstream of the annotated start site of *icmL* flanked by *BsaI* sites with ATTC and TATG overhangs for insertion into pBRM-P	This study
pICH41021-icmL$_{prom(129)}$	Derivative of pICH41021 containing 129 bp downstream of the annotated start site of *icmL* flanked by *BsaI* sites with ATTC and TATG overhangs for insertion into pBRM-P	This study
pICH41021-hrpB1$_{prom}$	Derivative of pICH41021 containing the promoter of *hrpB1* flanked by *BsaI* sites with ATTC and TATG overhangs for insertion into pBRM-P	Hausner et al., 2019
pICH41021-sfgfp	Derivative of pICH41021 containing *sfgfp* flanked by *BsaI* sites with TATG and GGTG overhangs for insertion into pBRM	Hausner and Büttner, unpublished
pBRM-P+T-virB2$_{prom}$-sfgfp	Derivative of pBRM-P+T encoding sfGFP under control of the putative *virB2* promoter	This study
pBRM-P+T-virB5$_{prom}$-sfgfp	Derivative of pBRM-P+T encoding sfGFP under control of the putative *virB5* promoter	This study
pBRM-P+T-virD4$_{prom}$-sfgfp	Derivative of pBRM-P+T encoding sfGFP under control of the putative *virD4* promoter	This study
pBRM-P+T-virG$_{prom}$-sfgfp	Derivative of pBRM-P+T encoding sfGFP under control of the putative *virG* promoter	This study
pBRM-P+T-dotA$_{prom}$-sfgfp	Derivative of pBRM-P+T encoding sfGFP under control of the putative *dotA* promoter	This study
pBRM-P+T-dotD$_{prom}$-sfgfp	Derivative of pBRM-P+T encoding sfGFP under control of the putative *dotD* promoter	This study

pBRM-P+T-icmL$_{prom(229)}$-sfgfp	Derivative of pBRM-P+T encoding sfGFP under control of the putative *icmL* promoter upstream of the annotated start codon	This study
pBRM-P+T-icmL$_{prom(129)}$-sfgfp	Derivative of pBRM-P+T encoding sfGFP under control of the putative *icmL* promoter downstream of the annotated start codon	This study
pBRM-P+T-hrpB1$_{prom}$-sfgfp	Derivative of pBRM-P+T encoding sfGFP under control of the *hrpB1* promoter	This study
pBRM-sfgfp$_{plac}$	Derivative of pBRM encoding sfGFP under control of the *lac* promoter	Hausner and Büttner, unpublished
pICH41021-virB2$_{prom}$	Derivative of pICH41021 containing the putative promoter of *virB2* flanked by *BpiI* sites with CTCA and CGAG overhangs for fusion with *dTALE-2*	This study
pICH41021-virB5$_{prom}$	Derivative of pICH41021 containing the putative promoter of *virB5* flanked by *BpiI* sites with CTCA and CGAG overhangs for fusion with *dTALE-2*	This study
pICH41021-virD4$_{prom}$	Derivative of pICH41021 containing the putative promoter of *virD4* flanked by *BpiI* sites with CTCA and CGAG overhangs for fusion with *dTALE-2*	This study
pICH41021-virG$_{prom}$	Derivative of pICH41021 containing the putative promoter of *virG* flanked by *BpiI* sites with CTCA and CGAG overhangs for fusion with *dTALE-2*	This study
pICH41021-dotA$_{prom}$	Derivative of pICH41021 containing the putative promoter of *dotA* flanked by *BpiI* sites with CTCA and CGAG overhangs for fusion with *dTALE-2*	This study
pICH41021-dotD$_{prom}$	Derivative of pICH41021 containing the putative promoter of *dotD* flanked by *BpiI* sites with CTCA and CGAG overhangs for fusion with *dTALE-2*	This study
pICH41021-icmL$_{prom(229)}$	Derivative of pICH41021 containing a putative promoter 229 bp upstream of the annotated start codon of *icmL* flanked by *BpiI* sites with CTCA and CGAG overhangs for fusion with *dTALE-2*	This study
pICH41021-icmL$_{prom(129)}$	Derivative of pICH41021 containing a putative promoter 129 bp downstream of the annotated start codon of *icmL* flanked by *BpiI* sites with CTCA and CGAG overhangs for fusion with *dTALE-2*	This study
pICH41021-hrpB1$_{prom}$	Derivative of pICH41021 containing the putative promoter of *hrpB1* flanked by *BpiI* sites with CTCA and CGAG overhangs for fusion with *dTALE-2*	This study
pAGM9121-virB2$_{prom}$	Derivative of pAGM9121 containing the putative promoter of *virB2* flanked by *BsaI* sites with GGAG and CGCT overhangs	This study
pAGM9121-virB5$_{prom}$	Derivative of pAGM9121 containing the putative promoter of *virB5* flanked by *BsaI* sites with GGAG and CGCT overhangs	This study
pAGM9121-virD4$_{prom}$	Derivative of pAGM9121 containing the putative promoter of *virD4* flanked by *BsaI* sites with GGAG and CGCT overhangs	This study
pAGM9121-virG$_{prom}$	Derivative of pAGM9121 containing the putative promoter of *virG* flanked by *BsaI* sites with GGAG and CGCT overhangs	This study
pAGM9121-dotA$_{prom}$	Derivative of pAGM9121 containing the putative promoter of *dotA* flanked by *BsaI* sites with GGAG and CGCT overhangs	This study
pAGM9121-dotD$_{prom}$	Derivative of pAGM9121 containing the putative promoter of *dotD* flanked by *BsaI* sites with GGAG and CGCT overhangs	This study
pAGM9121-icmL$_{prom(229)}$	Derivative of pAGM9121 containing the putative promoter 229 bp upstream of the annotated start codon of *icmL* flanked by *BsaI* sites with GGAG and CGCT overhangs	This study

pAGM9121-icmL$_{prom(129)}$	Derivative of pAGM9121 containing the putative promoter 129 bp downstream of the annotated start codon of *icmL* flanked by *BsaI* sites with GGAG and CGCT overhangs	This study
pAGM9121-hrpB1$_{prom}$	Derivative of pAGM9121 containing the putative promoter of *hrpB1* flanked by *BsaI* sites with GGAG and CGCT overhangs	This study
pICH47732-virB2$_{prom}$	Derivative of pICH47732 containing the putative promoter of *virB2* flanked by *BpiI* sites with TGCC and GCAA overhangs	This study
pICH47732-virB5$_{prom}$	Derivative of pICH47732 containing the putative promoter of *virB5* flanked by *BpiI* sites with TGCC and GCAA overhangs	This study
pICH47732-virD4$_{prom}$	Derivative of pICH47732 containing the putative promoter of *virD4* flanked by *BpiI* sites with TGCC and GCAA overhangs	This study
pICH47732-virG$_{prom}$	Derivative of pICH47732 containing the putative promoter of *virG* flanked by *BpiI* sites with TGCC and GCAA overhangs	This study
pICH47732-dotA$_{prom}$	Derivative of pICH47732 containing the putative promoter of *dotA* flanked by *BpiI* sites with TGCC and GCAA overhangs	This study
pICH47732-dotD$_{prom}$	Derivative of pICH47732 containing the putative promoter of *dotD* flanked by *BpiI* sites with TGCC and GCAA overhangs	This study
pICH47732-icmL$_{prom(229)}$	Derivative of pICH47732 containing the putative promoter 229 bp upstream of the annotated start codon of *icmL* flanked by *BpiI* sites with TGCC and GCAA overhangs	This study
pICH47732-icmL$_{prom(129)}$	Derivative of pICH47732 containing the putative promoter 129 bp downstream of the annotated start codon of *icmL* flanked by *BpiI* sites with TGCC and GCAA overhangs	This study
pICH47732-hrpB1$_{prom}$	Derivative of pICH47732 containing the putative promoter of *hrpB1* flanked by *BpiI* sites with TGCC and GCAA overhangs	This study
pICH77739-virB2$_{prom}$-dTALE2	Derivative of pICH77739 encoding dTALE-2 under control of the putative *virB2* promoter	This study
pICH77739-virB5$_{prom}$-dTALE2	Derivative of pICH77739 encoding dTALE-2 under control of the putative *virB5* promoter	This study
pICH77739-virD4$_{prom}$-dTALE2	Derivative of pICH77739 encoding dTALE-2 under control of the putative *virD4* promoter	This study
pICH77739-virG$_{prom}$-dTALE2	Derivative of pICH77739 encoding dTALE-2 under control of the putative *virG* promoter	This study
pICH77739-dotA$_{prom}$-dTALE2	Derivative of pICH77739 encoding dTALE-2 under control of the putative *dotA* promoter	This study
pICH77739-dotD$_{prom}$-dTALE2	Derivative of pICH77739 encoding dTALE-2 under control of the putative *dotD* promoter	This study
pICH77739-icmL$_{prom(229)}$-dTALE2	Derivative of pICH77739 encoding dTALE-2 under control of the putative *icmL* promoter	This study
pICH77739-icmL$_{prom(129)}$-dTALE2	Derivative of pICH77739 encoding dTALE-2 under control of the putative *icmL$_{new}$* promoter	This study
pICH77739-hrpB1$_{prom}$-dTALE2	Derivative of pICH77739 encoding dTALE-2 under control of the *hrpB1* promoter	This study

[1] Ap, ampicillin; Gm, gentamycin; Km, kanamycin; R, resistant; Rif, rifampicin; Sm, spectinomycin; Str, streptomycin.

References

Adlung, N., Prochaska, H., Thieme, S., Banik, A., Blüher, D., John, P., Nagel, O., Schulze, S., Gantner, J., Delker, C., Stuttmann, J., and Bonas, U. (2016). Non-host resistance induced by the *Xanthomonas* effector XopQ is widespread within the genus *Nicotiana* and functionally depends on EDS1. Front Plant Sci 7, 1796.

Battesti, A., and Bouveret, E. (2012). The bacterial two-hybrid system based on adenylate cyclase reconstitution in *Escherichia coli*. Methods 58, 325-334.

Bonas, U., Stall, R.E., and Staskawicz, B. (1989). Genetic and structural characterization of the avirulence gene *avrBs3* from *Xanthomonas campestris* pv. *vesicatoria*. Mol Gen Genet 218, 127-136.

Canteros, B.I. (1990). Diversity of plasmids and plasmid-encoded phenotypic traits in *Xanthomonas campestris* pv. *vesicatoria.* PhD thesis, University of Florida.

Figurski, D., and Helinski, D.R. (1979). Replication of an origin-containing derivative of plasmid RK2 dependent on a plasmid function provided *in trans*. Proc Natl Acad Sci USA 76, 1648-1652.

Hausner, J., Hartmann, N., Lorenz, C., and Büttner, D. (2013). The periplasmic HrpB1 protein from *Xanthomonas* spp. binds to peptidoglycan and to components of the type III secretion system. Appl Environ Microbiol 79, 6312-6324.

Hausner, J., Jordan, M., Otten, C., Marillonnet, S., and Büttner, D. (2019). Modular cloning of the type III secretion gene cluster from the plant-pathogenic bacterium *Xanthomonas euvesicatoria*. ACS Synth Biol 8, 532-547.

Kousik, C.S., and Ritchie, D.F. (1998). Response of bell pepper cultivars to bacterial spot pathogen races that individually overcome major resistance genes. Plant Disease 82, 181-186.

Kovach, M.E., Elzer, P.H., Hill, D.S., Robertson, M.A., Farris, M.A., Roop II, R.M., and Peterson, K.M. (1995). Four new derivatives of the broad-host-range cloning vector pBBR1MCS, carrying different antibiotic-resistance cassettes. Gene 166, 175-176.

Lorenz, C., Hausner, J., and Büttner, D. (2012). HrcQ provides a docking site for early and late type III secretion substrates from *Xanthomonas*. PLoS ONE 7, e51063.

Ménard, R., Sansonetti, P.J., and Parsot, C. (1993). Nonpolar mutagenesis of the *ipa* genes defines IpaB, IpaC, and IpaD as effectors of *Shigella flexneri* entry into epithelial cells. J Bacteriol 175, 5899-5906.

Murillo, J., Shen, H., Gerhold, D., Sharma, A., Cooksey, D.A., and Keen, N.T. (1994). Characterization of pPT23B, the plasmid involved in syringolide production by *Pseudomonas syringae* pv. *tomato* PT23. Plasmid 31, 275-287.

Otten, C., and Büttner, D. (2021). HrpB4 from *Xanthomonas campestris* pv. *vesicatoria* acts similarly to SctK proteins and promotes the docking of the predicted sorting platform to the type III secretion system. Cell Microbiol 23, e13327.

Schulze, S., Kay, S., Büttner, D., Egler, M., Eschen-Lippold, L., Hause, G., Krüger, A., Lee, J., Müller, O., Scheel, D., Szczesny, R., Thieme, F., and Bonas, U. (2012). Analyses of new type III effectors from *Xanthomonas* uncover XopB and XopS as suppressors of plant immunity. New Phytol 195, 894-911.

Szczesny, R., Jordan, M., Schramm, C., Schulz, S., Cogez, V., Bonas, U., and Büttner, D. (2010). Functional characterization of the Xps and Xcs type II secretion systems from the plant pathogenic bacterium *Xanthomonas campestris* pv. *vesicatoria*. New Phytol 187, 983-1002.

Van den Ackerveken, G., Marois, E., and Bonas, U. (1996). Recognition of the bacterial avirulence protein AvrBs3 occurs inside the host plant cell. Cell 87, 1307-1316.

Weber, E., Engler, C., Gruetzner, R., Werner, S., and Marillonnet, S. (2011). A modular cloning system for standardized assembly of multigene constructs. PLoS ONE 6, e16765.

Yanisch-Perron, C., Vieira, J., and Messing, J. (1985). Improved M13 phage cloning vectors and host strains: nucleotide sequences of the M13mp18 and pUC19 vectors. Gene 33, 103-119.

Table S2: Primers used in this study.

**Name**	**Sequence**[1]
**Primers for the generation of expression constructs**	
virD4-Bsa-for	TTT **GGTCTC**T *TATG* TCCAAACCCAAAATCATCG
virD4-950-rev	TTT **GGTCTC**T *AGGC* GAAGGTTTCATCCGCCTG
virD4-950-for	TTT **GGTCTC**T GCCT CGATCATGGGCACCTTG
virD4-1450-rev	TTT **GGTCTC**T *TGAC* GTTCTTCTTCTTGATGG
virD4-1450-for	TTT **GGTCTC**T *GTCA* CCCGAGGGCGGGAAACCAC
virD4-Bsa-rev	TTT **GGTCTC**T *CACC* CACTTTGATGGTAAGC
traI-pB-for	TTT **GGTCTC**T *TATG* ATGCTCAATGTGACGCCGATTCG
traI-pB-rev	TTT **GGTCTC**T *CACC* GCCAAAGCGTCCGTACTCGGACG
3751-Bsa-for	TTT **GGTCTC**T *TATG* CCGACGAACTACTCAC
3751-Bsa-rev	TTT **GGTCTC**T *CACC* ACCGACGCTGCGCCCCTGGC
XCV0332-for	TTT **GGTCTC**T *TATG* ACCGACGCACCGGTCATC
XCV0332-rev	TTT **GGTCTC**T *CACC* GCGCGGCGGCAAACGCAG
XCV1120-for	TTT **GGTCTC**T *TATG* GTGGAAGTCCGGCAAATC
XCV1120-rev	TTT **GGTCTC**T *CACC* CATTGTCATCCTTG
icmE-F1_for	TTT **GGTCTC**T *TATG* ATGGCTCAGCAGGAAAGCGATATC
icmE-F1-rev	TTT **GGTCTC**T *TCGT* CGGAGGCCGTACTTGCGAC
icmE-F2-for	TTT **GGTCTC**T *ACGA* CATGGCGGAAACCACCAG
icmE-F2-rev	TTT **GGTCTC**T *CGTC* CCGCGCATGACGCTTC
icmE-F3-for	TTT **GGTCTC**T *GACG* ATCGAAACCAGCGAGTTCG
icmE-F3-rev	TTT **GGTCTC**T *CACC* TTTGCCGAATGCGGATGC
dotB-for	TTT **GGTCTC**T *TATG* AACGCGGTGCTCTCCGACCAG
dotB-rev	TTT **GGTCTC**T *CACC* CTCACCCTCCCCGGCCAG
dotO-F1-for	TTT **GGTCTC**T *TATG* AAGATCGTCGAGCAAATCGCAGATATTG
dotO-F1-rev	TTT **GGTCTC**T *GTCT* TCGGTGTATTCGATGTCCAGGTAG
dotO-F2-for	TTT **GGTCTC**T *AGAC* GAAACCACCTTCGTGCTCAAG
dotO-F2-rev	TTT **GGTCTC**T *CACC* TGCACGGCGGTCACCTTG
dotL-F1-for	TTT **GGTCTC**T *TATG* AGCATCCAGGGCGTCGACAAGC
dotL-F1-rev	TTT **GGTCTC**T *CGGT* GGGGTCCTCAACCTTCCCAATG
dotL-F2-for	TTT **GGTCTC**T *ACCG* AAACCTGGGAGATGATCCAAGCAAT GGGCGGCGAAG
dotL-F2-rev	TTT **GGTCTC**T *CCGC* CGGCGCTGCCGCCACCAGATACAG
dotL-F3-for	TTT **GGTCTC**T *GCGG* TGGACTCAATGCTCAGCCGCAG
dotL-F3-rev	TTT **GGTCTC**T *CACC* AAGCTCATTCGGCGCTCCTGAACTG
virB10-for	TTT **GGTCTC**T *TATG* AGCGATCAGACCAACAAGCCGCAG
virB10-rev	TTT **GGTCTC**T *CACC* CGGGATGAACGTCACGCCGTCG
virB11-for	TTT **GGTCTC**T *TATG* TCGCAGATTGCCTCCTTC
virB11-rev	TTT **GGTCTC**T *CACC* CGCACCTGCATAGATG
XCVc0034-for	TTT **GGTCTC**T *TATG* CCTAAGGCCCTCTGGACATG
XCVc0034-rev	TTT **GGTCTC**T *CACC* GTGTCGCCATTGGTTGTCATCC
prom4361-for	TTT **GGTCTC**T *ATTC* AGGACAACGCGGTTCGTG
prom4361-rev	TTT **GGTCTC**T *CATA* GCGCGTGGCCTATGCG
Bsa-prom0160-for	TTT **GGTCTC**T *ATTC* TGATCGAAGATGCTGCTG
Bsa-prom0160-rev	TTT **GGTCTC**T *GATG* TGGACAGCGGCGCAG
Bsa-icmE-for-pLAND	TTT **GGTCTC**T *CATC* ATGATGGCTCAGCAGGAAAGC
Gent-Bsa-for	TTT **GGTCTC**T *TATG* TTACGCAGCAGCAAC
Gent-Bsa-Stop-rev	TTT **GGTCTC**T *CACC* TTAGGTGGCGGTACTTG

**Primers for colony PCR**	
dotC-detect-for	TTGATTTCGGCTCCTTGCTC
dotC-detect-rev	ATCTGGTCTGCCTGCTGCTC
dotB-detect-for	CTTCTGGTCCATGCCAAAGC
dotB_detect_rev	CGCGGAGGATGATCGAGATA
icmE-F1-for	TTT **GGTCTC**T *TATG* ATGGCTCAGCAGGAAAGCGATATC
icmE-F3-rev	TTT **GGTCTC**T *CACC* TTTGCCGAATGCGGATGC
virB11-detect-for	GAATCTCGATGACGGCGTTC
virB11-detect-rev	GAATCTCGCCCATGATGAC
virB4-detect-for	GTTCCAGCGCTCCCACGGTC
virB4-detect-rev	GTGCGCGTTGTCGAACACG
virB10-for	TTT **GGTCTC**T *TATG* AGCGATCAGACCAACAAGCCGCAG
virB10-rev	TTT **GGTCTC**T *CACC* CGGGATGAACGTCACGCCGTCG
virD4-detect-for	GGAGAACTTCGACCTGACCA
virD4-detect-rev	GTCCGTGAGCAGGAAGTCAT
avrBs3-Bsa-for	TTT **GGTCTC**T *TATG* GATCCCATTCGTTC
avrBs3-Bsa-50 rev	TTT **GGTCTC**T *GATC* GGACATCGTCCGCCGAGC
hpaF-seq-for	CTTCAATTGACGATGGTC
hpaG-seq-rev	CTGCATGAGGCCGATGTTTTCAG
pKT25/pUT18-for	ACTGGAAAGCGGGCAGTGAG
pKT25-rev	GCTGCGCAACTGTTGGGAAG
pUT18-rev	GGTGTCGGGGCTGGCTTAAC
pBRM-1180-for	ATGCATGCGCCCAATACGCAAACCG
pBRM-endlacZ-rev	CACTCATCGCAGTCGGCCTATTG
M13-for	GTAAAACGACGGCCAGTG
M13-rev	GGAAACAGCTATGACCATG
pOK-mcs-for	CAAACCACGTCAAATAATCAATTATG
pOK-mcs-rev	CTTGTCCTTTAACGAGGATTGTTAC
**Primers for the generation of deletion constructs**	
pXCV183-F1-for	TTT **GGTCTC**T *CGAC* CCGGACAGCTCGGTTCCGGCCTTG
pXCV183-F1-rev	TTT **GGTCTC**T *ACTG* ATTGCACCATGCAACTAGCCCCG AGCTGAAG
pXCV183-F2-prom-for	TTT **GGTCTC**T *CAGT* TGACATAAGCCTGTTCGGTTCG
pXCV183-F2-prom-rev	TTT **GGTCTC**T *CGTT* GCTGCTCCATAACATCAAACATCG
pXCV183-F3-kan-for	TTT **GGTCTC**T *AACG* ATGATTGAACAAGATGGATTGCA CGCAG
pXCV183-F3-kan-rev	TTT **GGTCTC**T *GAAT* TCAGAAGAACTCGTCAAGAAGGC GATAGAAG
pXCV183-F4-promlacZ-for	TTT **GGTCTC**T *ATTC* GCAGCTGGCACGACAG
pXCV183-F4-GFP-rev/	AAA **GGTCTC**T *CACC* TCATCATTTGTACAG
pXCV183-F5-for	TTT **GGTCTC**T *GGTG* TCAGGGCGCATCGGTATCGCCACGAG
pXCV183-F5-rev	TTT **GGTCTC**T *ATGG* GCCGCAGCCGATACTAAGCACCTAGAG
virG-pOGG2-1-for	TTT **GGTCTC**T *CGAC* CCTGGTGGGACACTCCTGGGG CGGCAC
virG-pOGG2-1-rev	TTT **GGTCTC**T *GGAA* TGCAGCTTGATCATCGACTCTCTATC
virG-pOGG2-2-for	TTT **GGTCTC**T *TTCC* GAGCACGCTCGATGACACAGCCGCC GGCGCGAGC
virG-pOGG2-2-rev	TTT **GGTCTC**T *ATGG* AGCAGCACGGCGCTGAAGATCGTC
pOGG2-virA/C-for	TTT **GGTCTC**T *CGAC* TGGAACCAGCAGTTCCAGCACG
pOGG2-virA/C-rev	TTT **GGTCTC**T *GTGC* CTAGAAGTTACAACGCAGGATG
pOGG2-virA/N-for	TTT **GGTCTC**T *GCAC* AGGCTGAAGAAGATCAGC
pOGG2-virA/N-rev	TTT **GGTCTC**T *ATGG* CGAGCCAGGCCAGACCATCG
virB6-10-pOGG2-1-for	TTT **GGTCTC**T *ATGG* TTGCAACAGCAGTTTCAGCAG
SD-virB6-10-pOGG2-1-rev	TTT **GGTCTC**T *TGGT* TGTCATCCCCATAAACGTGCTTG

virB6-10-pOGG2-2-for	TTT **GGTCTC**T *ACCA* CGTGACGTTCATCCCGTGAG
virB6-10-pOGG2-2-rev	TTT **GGTCTC**T *CGAC* GGCCAACTTCTCAAACGGCAGC
virB4-pOGG2-Bsa-FIfor	TTT **GGTCTC**T *CGAC* CGCTTGGACATAGCTGTG
virB4-pOGG2-Bsa-FIrev	TTT **GGTCTC**T *GTCA* GTCGAACTTCTCTTTTAAAACCTC
virB4-pOGG2-Bsa-FIIfor	TTT **GGTCTC**T *TGAC* CATGAAAAAGACCC
virB4-pOGG2-Bsa-FIIrev	TTT **GGTCTC**T *ATGG* CTATACCGCGAGCGCAAG
virD4-FII-BsaI-rev	TTT **GGTCTC**T *CGAC* CGTCGCGGGTACTTGAG
virD4-FII-BsaI-for	TTT **GGTCTC**T *TGAT* TGATGTCACATTTGCAG
virD4-FI-BsaI-for	TTT **GGTCTC**T *ATCA* GCGCCCTTCTCCTTAGG
virD4-FI-BsaI-rev	TTT **GGTCTC**T *ATGG* TGGCATGTGTCAGTTCG
virB11-pOGG2-Bsa-FII-for	TTT **GGTCTC**T *CGAC* CATGAAGTCGGCCATCATC
virB11-pOGG2-Bsa-FII-rev	TTT **GGTCTC**T *GTCA* GGCTCGGCCCTCACGGGATG
virB1-pOGG2-Bsa-FI-for	TTT **GGTCTC**T *TGAC* CGGCTTTTCTCGCATGC
virB1-pOGG2-Bsa-FI-rev	TTT **GGTCTC**T *ATGG* GCCATCGTGTTGCGAACCTT
pOGG2-icmE-F1-for	TTT **GGTCTC**T *CGAC* CCTGCCGGTACCACTGTGC
pOGG2-icmE-FI-rev	TTT **GGTCTC**T *TTTC* CTGCTGAGCCATCACG
pOGG2-icmE-FII-for	TTT **GGTCTC**T *GAAA* TAAAGGCTCCCCATGAAC
pOGG2-icmE-FII-rev	TTT **GGTCTC**T *ATGG* CGCTTTCAGTTGGATAC
pOGG2-dotCB-F1-for	TTT **GGTCTC**T *CGAC* ATGTTTGCGCCCGATAGC
pOGG2-dotCB-FI-rev	TTT **GGTCTC**T *TCAC* AAACCGCCATTGCTG
pOGG2-dotCB-FII-for	TTT **GGTCTC**T *GTGA* GAGGGTGAGTGATGTCAG
pOGG2-dotCB-FII-rev	TTT **GGTCTC**T *ATGG* GCGATCTGCGGTTACCAG
**Primers for the amplification of predicted promoter regions**	
promvirB2-for	TTT **GGTCTC**T *ATTC* ACTATGCGGTTTATCTGGTTG
promvirB2-rev	TTT **GGTCTC**T *CATA* TCTGAAACCTCTCAGATTGTTAG
promvirB5-for	TTT **GGTCTC**T *ATTC* ATTGCCGGACTGACGCACATCTATCG
promvirB5-rev	TTT **GGTCTC**T *CATA* ATCGAAGGTCGGGATGGGGGTTCG
promvirD4-for	TTT **GGTCTC**T *ATTC* GATATCGTGTTGTGGCGATTACG
promvirD4-rev	TTT **GGTCTC**T *CATA* GCGCCCTTCTCCTTAGGCTTG
promvirG-for	TTT **GGTCTC**T *ATTC* GTCGGATCGGGCTGGCGACG
promvirG-rev	TTT **GGTCTC**T *CATA* CGACTCTCTATCCCGGCATG
promdotA-for	TTT **GGTCTC**T *ATTC* GCCTGATCTGGCCCGGTCATC
promdotA-rev	TTT **GGTCTC**T *CATA* ATTGACGAGCGCGCCACAAACG
promdotD-for	TTT **GGTCTC**T *ATTC* TAGCGACACATATACGTGACCGTG
promdotD-rev	TTT **GGTCTC**T *CATA* CGGGGCACGTTGCCTTTCGTTG
promicmL-for	TTT **GGTCTC**T *ATTC* CCGGCATAGGTTGGACACAGG
promicmL-rev	TTT **GGTCTC**T *CATA* GAACTCTGCCGGTCACTCG
promicmL(129)-for	TTT **GGTCTC**T *ATTC* GTGCAATCGGTGTCCAACTATGC
promicmL(129)-rev	TTT **GGTCTC**T *CATA* TCGTCGCGCCACAACTGTTTG
dTALE-pvirB2-for	**GAAGAC** AA *CTCA* GGAG ACTATGCGGTTTATCTGGTTG
dTALE-pvirB2-rev	**GAAGAC** AA *CTCG* AGCG TCTGAAACCTCTCAGATTGTTAG
dTALE-pvirB5-for	**GAAGAC** AA *CTCA* GGAG ATTGCCGGACTGACGCACATCTATCG
dTALE-pvirB5-rev	**GAAGAC** AA *CTCG* AGCG ATCGAAGGTCGGGATGGGGGTTCG
dTALE-virD4-for	**GAAGAC** AA *CTCA* GGAG GATATCGTGTTGTGGCGATTACG
dTALE_virD4_rev	**GAAGAC** AA *CTCG* AGCG GCGCCCTTCTCCTTAGGCTTG
dTALE-pvirG-for	**GAAGAC** AA *CTCA* GGAG GTCGGATCGGGCTGGCGACG
dTALE-pvirG-rev	**GAAGAC** AA *CTCG* AGCG CGACTCTCTATCCCGGCATG
dTALE-pdotA-for	**GAAGAC** AA *CTCA* GGAG GCCTGATCTGGCCCGGTCATC
dTALE-pdotA-rev	**GAAGAC** AA *CTCG* AGCG ATTGACGAGCGCGCCACAAACG
dTALE-pdotD-for	**GAAGAC** AA *CTCA* GGAG TAGCGACACATATACGTGACCGTG
dTALE-pdotD-rev	**GAAGAC** AA *CTCG* AGCG CGGGGCACGTTGCCTTTCGTTG

dTALE-picmL-for	**GAAGAC** AA *CTCA* GGAG CCGGCATAGGTTGGACACAGG
dTALE-picmL-rev	**GAAGAC** AA *CTCG* AGCG GAACTCTGCCGGTCACTCG
dTALE-picmL(129)_for	**GAAGAC** AA *CTCA* GGAG GTGCAATCGGTGTCCAACTATGC
dTALE-picmL(129)-rev	**GAAGAC** AA *CTCG* AGCG TCGTCGCGCCACAACTGTTTG
dTALE-phrpB1-for	**GAAGAC** AA *CTCA* GGAG GAATGTGTGGATGGCCT GGCGAAG
dTALE_phrpB1-rev	**GAAGAC** AA *CTCG* AGCG GCGGACCGTACACGTGGGGAACG
[1] *Bsa*I and *Bpi*I recognition sites are shown in bold, resulting overhangs in italics.	

Table S3: Accession numbers and predicted functions of VirB/VirD4 proteins from *A. tumefaciens* and *x. axonopodis* pv. *citri* strain 306.

Protein	***A. tumefaciens*** **strain GV3101** [NZ_KY000036.1]		***X. axonopodis*** **pv.** ***citri*** **strain 306** Chromosome [AE008923]		***X. axonopodis*** **pv.** ***citri*** **strain 306** pXAC64 [AE008925]		**Putative function/ localization[2]**
	accession number[1]	aa	accession number[1]	aa	accession number[1]	aa	
VirB1	WP_010974915.1	245	AAM37466.1	280	AAM39282.1	292	lytic transglycosylase
VirB2	WP_010891502.1	121	AAM37465.1	136	AAM39293.1	125	pilin subunit
VirB3	WP_010891501.1	108	AAM37464.1	103	AAM39292.1	99	inner membrane platform
VirB4	WP_010974916.1	789	AAM37463.1	817	AAM39291.1	877	ATPase
VirB5	WP_010891499.1	220	AAM37462.1	275	AAM39290.1	220	pilus-tip protein
VirB6	WP_010974917.1	295	AAM37461.1	350	AAM39287.1	288	inner membrane platform
VirB7	WP_010891497.1	168	AAM37471.1	139	AAM39288.1	131	lipoprotein, outer membrane core complex
VirB8	WP_010891496.1	237	AAM37470.1	348	AAM39286.1	224	inner membrane platform
VirB9	WP_010891495.1	393	AAM37469.1	255	AAM39285.1	260	outer membrane core complex
VirB10	WP_010891494.1	377	AAM37468.1	389	AAM39284.1	406	inner/outer membrane core complex
VirB11	WP_010974918.1	344	AAM37467.1	346	AAM39283.1	340	ATPase
VirD4	WP_010974920.1	668	AAM37472.1	557	-	-	ATPase
VirG	WP_010891492.1	253	AAM36537.1	124	-	-	two-component system response regulator
VirA	WP_010974914.1	833	AAM36536.1	676	-	-	two-component system sensor kinase
TraG	-	-	-	-	AAM39276.1	524	conjugative coupling protein TrwB
TraI	-	-	-	-	AAM39277.1	991	conjugative relaxase TrwC

[1] These proteins were used for the pairwise sequence alignments with corresponding proteins from *X. euvesicatoria* strain 85-10. The corresponding amino acid identities between homologous proteins are given in Fig. 1.
[2] See main text for details and references.

Table S4: Sequences of predicted *vir* and *icm/dot* promoters from *X. euvesicatoria*.

Predicted promoter of:	Sequence (5' – 3')[1]
virB2	actatgcggtttatctggttgaggcggagcacgagagcagccggtggtggcggatgacggtgaagttacccacgcacaacaaag<br>cttatgaggtcgttaccgcgcttggtaaaaccaagctctggaaacgcttggacatagctgtggatttcatcaaggagagctgcccgc<br>acacaaagaccgtgttcgtagtttttgccccgtagcacacctaacaatctgagaggtttcaga
virB5	attgccggactgacgcacatctatcgctactttgcctcacatcgccaattggcaacccaagggcctttacgcagacaaggtcctaag<br>attgggagaaatgatcagtgcccatgtggcagtgggcggaagtacaagcattgctgtgctaccagcgctccgacatttcattgatac<br>cggcttaactgacttccctgttccattgctccccgaacccccatcccgaccttcgat
virD4	gatatcgtgttgtggcgattacgcgtgacgaggtgtatgcacccgacgcggtcgtggcatacgccgtcgtgaccgatgcgggtacg<br>cggatcacgccggatttgtctctggaccaggccaaggtctggatcgattcgctggtggagagcgagagcggcggacgcaagtcc<br>gagcttgtcgaccataagccggtcgtgcgtcgctagcccgccatcgtgatgcaagcctaaggagaagggcgc
virG	gtcggatcgggctggcgacgggatcggtccctcacccgtgttgtcgcagagcgagtggctggtcacaagatcgaacaaaagtcgt<br>catcgaccacggcacggagcgtgaccacccgctcgtcgacgtggcaagccgacacaaaaacaccctagcatcgtgcgtgaagat<br>gccgtccccgcgacggcgtatcgtggcacgccgcgcatggacgcacttccccaacgcggtcgattcggtcaccccagccgatcg<br>acactcggaatctgtccgcgaacgcggtcatgccgggatagagagtcg
dotA	gcctgatctggcccggtcatccggcggccgagcgcgaccaggcgatcggctaagagtggctcccgatccacagtccagttctcg<br>gccctatccatgacgctgaccaagtcccccagttccggggcataactacgccagaactcgacgagcttggggggtgcgaggtcta<br>gcgcagccatgtcacatccgtcactatatgttatgccttagtttaacatcgtttgtggcgcgctcgtcaat
dotD	tagcgacacatatacgtgaccgtgcacagagccggcctgtcacattcgttcgcatatatgcgacgcgagtacacggtggaagtgtc<br>ggtgccgagtggtgatgtgatgctgtggggggttgattgcagagatacggcgggataacctgcgccacaaatgttgtgtaatccatg<br>ccacgtattcaccaacgaaaggcaacgtgccccg
icmL$_{229}$	ccggcataggttggacacaggccgccgaacagctgatttctagtgattttgcgcggtgcttgccgacgtccatatcacttggtgcggt<br>aagtcagtgaagtgatgaccgctgtagcactcgcgcgataagtagcgccggcgctacatcgtggaatggacgtccagcagctgct<br>gatgtgcgccggttgtgaccgcataaaggcgcccggccgagtgaccggcagagttc
icmL$_{129}$	gtgcaatcggtgtccaactatgccgttcttcccacggccaaaggccacattcgttggcatatatcgttgacccgcctgttatggtgtcgt<br>atcctcgtgaacgatttccaaacagttgtggcgcgacga

[1] Predicted *vir* boxes are indicated in yellow.

Table S5: Accession numbers and predicted functions of Icm/Dot proteins from *L. pneumophila.*

Protein	*L. pneumophila* strain *Philadelphia1* [NC002942.5]		Putative function/ localization[2]
	accession number[1]	aa	
DotA	WP_010948386.1	1048	inner membrane protein
DotB	WP_011947587.1	369	ATPase
DotC	WP_010948375.1	303	lipoprotein, outer membrane core complex
DotD	WP_010948374.1	163	lipoprotein, outer membrane core complex
IcmT	WP_010946190.1	86	inner membrane protein
IcmW	WP_010948388.1	151	cytoplasmic chaperon
IcmP/DotM	WP_011214699.1	376	inner membrane protein
IcmO/DotL	WP_010946195.1	783	ATPase
IcmL/DotI	WP_010946198.1	212	inner membrane protein
IcmK/DotH	WP_015444826.1	360	periplasmic
IcmE/DotG	WP_010946200.1	1048	inner membrane protein
IcmG/DotF	WP_010946201.1	269	inner/outer membrane core complex
IcmC/DotE	WP_010946202.1	194	inner membrane protein
IcmD/DotP	WP_011214707.1	132	inner membrane protein
IcmJ/DotN	WP_011214708.1	208	cytoplasmic
IcmB/DotO	WP_010946205.1	1009	cytoplasmic

[1] These proteins were used for the pairwise sequence alignments with corresponding proteins from *X. euvesicatoria* strain 85-10. The corresponding amino acid identities between homologous proteins are given in Fig. 2.
[2] See main text for details and references.

Table S6: Transfer of plasmid pXCV38$_{82\text{-}8}$::*spec*^{R} from strain 82-8 into the recipient strain 85-10::*gent*^{R}.

**Donor**		**Recipient**		**Transconjugant**[1]		
Strain	CFU	Strain	CFU	Strain	CFU	% of recipient
82-8 pXCV38$_{82\text{-}8}$::*spec*^{R}	5×10^8	85-10::*gent*^{R}	5×10^8	85-10::*gent*^{R} pXCV38$_{82\text{-}8}$::*spec*^{R}	3.59×10^5	0.072
	6.45×10^8		6.45×10^8		1.343×10^7	2.08
	7.05×10^8		7.05×10^8		8.23×10^6	1.17
85-10::*gent*^{R} pXCV38$_{82\text{-}8}$::*spec*^{R}	5.55×10^8	85-10::*kan*^{R}	5.55×10^8	85-10::*kan*^{R} pXCV38$_{82\text{-}8}$::*spec*^{R}	44.000	0.008
	5.52×10^8		5.52×10^8		41.500	0.008
	4.56×10^8		4.56×10^8		35.600	0.008

[1] Transconjugants were selected on NYG plates containing rifampicin, gentamycin and spectinomycin. The presence of plasmid pXCV38$_{82\text{-}8}$::*spec*^{R} was confirmed by agarose gel electrophoresis and PCR (see text). Numbers represent the numbers of transconjugants obtained in three independent conjugation experiments using the listed donor and recipient strains.

Table S7: XVIPCDs in T4S candidate substrates from *X. euvesicatoria.*

Protein	Amino acid sequence[1]
XCV0332	MTDAPVIHQGPVGPDHPEHPDHYLFAQIREAVSVLDAELGKPVDQASERMAARLLPLA<br>KQHGFDQVDYVVLSRHLGEVGENVFLVRGELSDPAHLRAHITTQEAMETSVDASLAQ<br>LEEINRRLMLRLPPR
XCV1120	MEVRQIGKGRAGQPDIEPQAVASNEAVLADNPAHPDHSTYQQIHSWVRGTGNWNEEE<br>SKNVTASLYKQQTEDPLLKRVDQVTGGLGRDGAHNVFAVYAPHGMGVAPMFHAHV<br>DGRQAAQEPAQQNLQQAEAIQQTQVRQQVLEQTQQQNVQQEQGPRMTM
XCV3751	MPTNYSRAQVLDIIEREAAERNIPRDDFLRFAHVETGGAFDELASRGPGGAKGLFQFTP<br>PTAQQYGIAGRELDAVANTDAAARLYLDNRTSLVNRHERDGRPYLSGKPEPDGLDMY<br>LAHQQGAGGYRSLQTAIATGSFGLESTRANILNNVGEKELKSLTGVDSATFRRMSDKD<br>MAQTFVQYWDTKMDRIRIPEKGIEPISTTQQAPSHAAPAHSAPAQPEKPAAQGIALHAA<br>YDLTNKYDHVKYGLGAKDPDKGRVDCSGWVVEMQNATMDEINKGAGKAIFTKDEKF<br>SPGFDSASELLRKAELRSGVLIQGKDVTAQTLKEGMIIGEDNGPQSWDKGRYKGIDHIT<br>MVVRDPKDGQLKISQSRGGEGVELSSLDSYLERKHAKGVKLYASDPLSEARDLLQERS<br>QNKQQSHAATEHKPSQAHAAADAPGVLRENARGAEVRTLQQTLQQLGYKDAGGNEL<br>KADGAYGQRTSEAVKAFQRAHGLQDDGVVGRDTQAALKQAEKTPLLSEKTNPDHPLF<br>NQAVNKLEQLGPNAFANRQQLVNAAGHMTFEAKVSGMQRIDMVAQSKDGNGLFAV<br>QGQPTDPAHQRIYTDKATAAERPLEQSSNAVRQEAQTQTQVQDQHEQQRSQGRSVG
TraI	MLNVTPIRGNNQYAAAHYFSAADDYYAKEHPGEWQGQGAQALGLTGPVEQAQLSRL<br>LDGQLPNGERIQTTFDPTDNKKRMGLDLTFSAPKSVSMQALVAGDKDVTAAHDRAVT<br>RALEQVERLAEARKKVKGKSYRERTANMVIGKFRHEMSRAKDPQLHTHAVVLNMTQ<br>RADGAWRALSNEDIFRVQHEVDALYKAELARGLQALGYAIRLVDDQGNFELDHISRD<br>QIEAFSARSRVIEEALANEGKTRATATTLEKQIISLATRPRKDESDRELVKQYWVEKSRE<br>LGIDYGLRSQLDGRTYEAGDSFGTRGRERGDAGDRIAATSLPASLTPAQAVVQYAINH<br>LTEREAVVRETALTATALRRAVGLAGPDEVRAEIKRLAGQGALIEAVPAYRMADRKD<br>GPALSSAGWKSYLQELKGWSDKQAQQYVGNAIKQGSLVPAEKRYTTQKALAREKAIL<br>AIERTGRGAIEPIMTAAAVKTALEGTALSAGQRSAVETIVSTKNRFVGIQGDAGTGKTY<br>TVNQAVALIKQASAVSEGYRTVALAPYGNQVKALKNEGLEAHTLASFLRTKDKPIDGK<br>TIIVLDESGVVGARQMEQVMRVVEKAGARMVLLGDTKQTEAIEAGKPFAQLQQDGM<br>QTARISEIQRQKDHELKTAVEHAAEGRVTPSLAHIKHVEELKEPIERHRAIVNDYIQLTE<br>PERRETLIVAGTNEARREINRMVRQSLDLTGKGREFETLTRVDMTQAQRRFAPSYQPG<br>MVIQPEKDDQKAGLTRGETYRVKDALPGNALVLQRQDGTTTTINPRKATQLSVYRLER<br>AELSVGDTVRINRNDPGRDLTNGDRMRVAGVIGGVVKLESVEQRDGRPARALELPTN<br>RPLHLEHAYASTVHSAQGLTNDRALIALDTNSRTTSMNLYYVAISRACHEARVYTNSV<br>KELPAAIARRFDKTTALAIQRERQLQRRDAGMQPKGAADGKQALQRQQFQQQRKQPA<br>SGKKPSEYGRFG

[1]Conserved amino acid sequences of the XVIPCD are indicated as follows: GLxRIDHV in yeqllow, FAVQGxxDPAHxRAHV in green and the glutamine-rich C-terminal tail in purple.

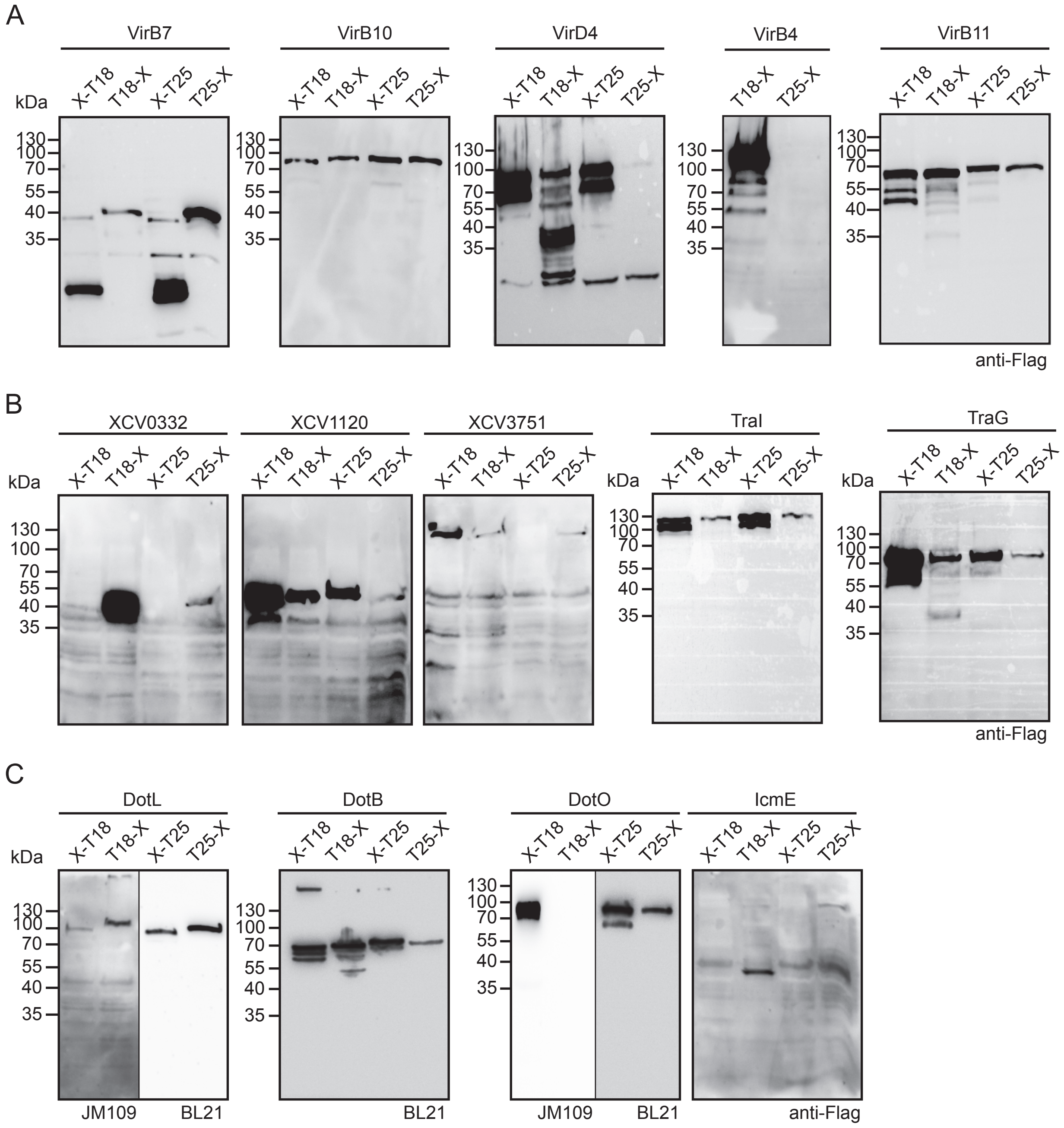

Figure S1
Drehkopf et al.

Figure S1: Synthesis and immunological detection of T18 and T25 fusion proteins in *E. coli* strain JM109.
A) Immunological detection of VirB fusion proteins. Cell extracts from *E. coli* strain JM109 with expression constructs encoding T18 and T25 fusions of VirB7, VirB10, VirB4, VirB11 and VirD4 as indicated were analysed by immunoblotting, using a FLAG epitope-specific antibody. Asterisks mark signals at the expected size of the fusion proteins. Additional signals likely correspond to degradation products or result from unspecific binding of the antibody to proteins in the cell extracts.
B) Analysis of T18 and T25 fusions of candidate T4S substrates and TraG. Cell extracts of *E. coli* strain JM109 containing expression constructs for the synthesis of T18 and T25 fusions of XCV0332, XCV1120, XCV3571, TraI and TraG as indicated were analysed by immunoblotting as described in (A). Proteins at the expected size are indicated by asterisks. Note that XCV0332-T18, XCV0332-T25 and XCV3751-T25 were not detectable.
C) Immunological detection of T18 and T25 fusions of Icm and Dot proteins. JM109 cell extracts containing expression constructs for the synthesis of T18 and T25 fusions of DotL, DotB, DotO and IcmE were analysed by immunoblotting as described in (A). Proteins at the expected size of the fusions are indicated with asterisks. Additional signals likely correspond to degradation products or protein complexes in case of DotB, which were not dissolved during SDS-PAGE. Note that T18-DotO and IcmE-T18 were not detectable.

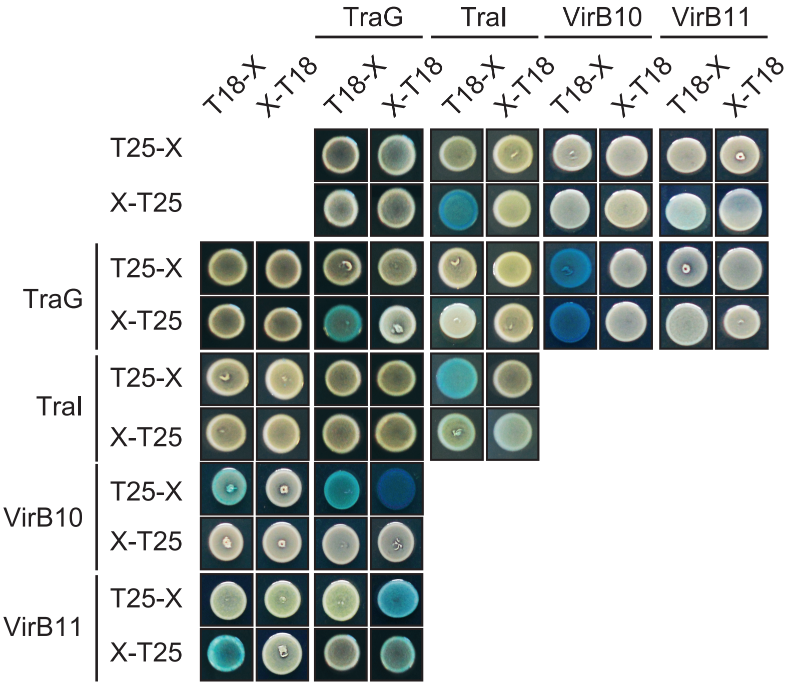

Figure S2
Drehkopf et al.

Figure S2: Results of BACTH assays with TraG from *X. euvesicatoria.*
T18 and T25 fusions of TraG, the predicted ATPase VirB11, the structural component VirB10 and the putative relaxase TraI were analysed in *E. coli* BTH101 cells as indicated and bacterial cultures were grown on indicator plates containing X-gal and IPTG. As control, fusion proteins were tested against the T18 or T25 domain alone as indicated. All proteins were stably synthesized as was shown by immunoblot analysis of bacterial cell extracts, using a FLAG epitope-specific antibody (see Fig. S1). Interaction studies were performed at least three times with similar results. One representative colony is shown. Results are summarized in Table 2.
